# ﻿A taxonomic revision of Garcinia section Discostigma (Clusiaceae) in Thailand

**DOI:** 10.3897/phytokeys.261.156445

**Published:** 2025-08-22

**Authors:** Chatchai Ngernsaengsaruay, Pichet Chanton, Weereesa Boonthasak, Wanwisa Bhuchaisri

**Affiliations:** 1 Department of Botany, Faculty of Science, Kasetsart University, Chatuchak, Bangkok 10900, Thailand; 2 Biodiversity Center, Kasetsart University (BDCKU), Chatuchak, Bangkok 10900, Thailand; 3 Suan Luang Rama IX Foundation, Nong Bon Subdistrict, Prawet District, Bangkok, 10250, Thailand; 4 Royal Park Rajapruek, Highland Research and Development Institute (Public Organization), Chiang Mai 50100, Thailand; 5 Chatuchak, Bangkok 10900, Thailand

**Keywords:** Dioecy, exudate-containing canals, glandular wavy lines, Guttiferae, lectotypi­fications, Malpighiales, synonymisations, taxonomy

## Abstract

Garcinia section Discostigma (Clusiaceae) is revised for Thailand with four species: *G. merguensis*, *G. minutiflora*, *G. rostrata*, and *G. santisukiana*. Morphological descriptions, illustrations, and an identification key to the species are provided, along with notes on distributions, habitats and ecology, phenology, preliminary conservation assessments, etymology, vernacular names, uses, and specimens examined. Two taxa, *Garcinia brevirostris* and *G. calophylla*, are newly synonymised under *G. rostrata*. Nine names are lectotypified here, including four associated synonyms of *Garcinia merguensis*–G. merguensis var. truncata, G. merguensis var. pyramidata, *G. fulva*, and *G. lanceolata*—as well as *G. rostrata* and four associated synonyms of *G. rostrata*: *G. brevirostris*, *G. calophylla*, *G. gitingensis*, and *G. wrayi*. Two species, *Garcinia minutiflora* and *G. santisukiana*, have a conservation status of Vulnerable [VU], while the other two species, *G. merguensis* and *G. rostrata*, have a conservation status of Least Concern [LC].

## ﻿Introduction

*Garcinia* L. is a group of evergreen trees, occasionally shrubs, which are usually dioecious but sometimes polygamo-dioecious. It also includes obligately and facultatively agamospermous species (Ngernsaengsaruay et al. 2023a, b). The colour of exudate secreted from cut boles, twigs, leaves, and fruits can be yellow, pale yellow, white, cream, or clear (Ngernsaengsaruay et al. 2022a). The genus includes c. 400 species (Gaudeul et al. 2024; POWO 2025) and is the largest genus in the Clusiaceae Lindl. (Guttiferae Juss.). It is a pantropically distributed genus and has centres of diversity located in Africa (Madagascar), Australasia, and Southeast Asia (Sweeney and Rogers 2008; Gaudeul et al. 2024). In Asia, *Garcinia* is most diverse in the Malesian Region but also spreads north into southern China, west to India, and east to the Micronesian islands (Nazre et al. 2018).

A worldwide sectional treatment of *Garcinia* was presented by Jones (1980) in an unpublished Ph.D. thesis in which the genus was classified into 14 sections based mainly on floral morphology, especially male flowers and pollen morphology. The latest is an updated infrageneric classification of the genus *Garcinia* proposed by Gaudeul et al. (2024), who recovered nine major clades falling within two major lineages and recognised 11 sections. Jones (1980) treated Garcinia sect. Discostigma (Haask.) Hook. f. subsection Discostigma, using Pierre’s (1882, 1883) sectional name, and recognised 53 species in this subsect. (Table 1). Two taxa representing Garcinia sect. Discostigma–*G. eugeniifolia* Wall. ex T. Anderson and *G. rostrata* (Hassk.) Miq.–constitute clade 4 (Sweeney 2008). Jones’s (1980)Garcinia sect. Discostigma contains many species with floral morphology like that found in *G. eugeniifolia* and *G. rostrata*. Sweeney (2008) noted that there were two groups of species placed into Garcinia sect. Discostigma by Jones (1980) that differed from typical members of the section by their androecial morphology. One group of species, including *Garcinia balansae* Pierre, *G. lanessanii* Pierre, *G. terpnophylla* Thwaites, and *G. warrenii* F. Muell., differs by having their stamens fused to the petals. The position of *Garcinia warrenii* in the trees presented by Gaudeul et al. (2024) and in Sweeney (2008) suggests that some of these species may be better placed within G. sect. Macrostigma Pierre (clade 9). However, Gaudeul et al.’s (2024) molecular analyses find strong support for placement of *Garcinia balansae* within G. sect. Discostigma. The second group of species includes *Garcinia dives* Pierre, *G. hunsteinii* Lauterb., *G. linii* C. E. Chang, *G. luzoniensis* Merrill, and *G. palawanensis* Elmer and is restricted to New Guinea, the Philippines, and Taiwan (Jones 1980). This latter group is reported to have peltate anthers, like species of Garcinia sect. Hebradendron Planch. & Triana (sensu Jones 1980); nevertheless, Jones (1980) placed them into G. sect. Discostigma because they share the same stamen arrangement and pollen apertures as typical members of the section. Unfortunately, species representing the *Garcinia dives* Pierre group have not yet been included in molecular phylogenetic analyses (Sweeney 2008).

Garcinia sect. Discostigma was recently updated by Gaudeul et al. (2024), who recognised 60 species (Table 1). Species in this section are distributed in Indomalaya, tropical Australasia, and Oceania (Gaudeul et al. 2024). The section is distinguished by its flowers with four sepals and four petals; male flowers with a pistillode; stamens arranged into four fascicles that are distally covered with sessile to subsessile two-thecous anthers; bilocular ovaries (or unilocular; four-locular in *Garcinia yunnanensis* Hu); unlobed and smooth stigmas; fruits with a smooth surface and capped with a conspicuous discoid stigma; caducous sepals in fruits; and terminal or axillary inflorescences with few to many flowers (Gaudeul et al. 2024).

In Thailand, the genus *Garcinia* was enumerated by Craib (1925) with 20 species. Gardner recorded six species in northern Thailand (Gardner et al. 2000) and 23 species (including five unidentified species) in Peninsular Thailand (Gardner et al. 2015). A taxonomic revision of *Garcinia* in Thailand has recently been undertaken by the first author as part of the Flora of Thailand project, and treatments for several sections have been produced (Ngernsaengsaruay and Suddee 2016, 2022; Ngernsaengsaruay 2022; Ngernsaengsaruay et al. 2022a, b, 2023a, b, 2024a, b; Ngernsaengsaruay and Chanton 2024; Ngernsaengsaruay et al. 2025).

Based on these publications, c. 30 accepted species of the genus have been reported in Thailand, belonging to seven sections, along with some unplaced species. However, identifications mostly rely on the literature, and this is the case for Garcinia sect. Discostigma, which has never been revised for Thailand. Therefore, in this paper, we provide an updated account for this section in Thailand in order to present a taxonomic treatment that includes synonymisations, lectotypifications, morphological descriptions, and illustrations, along with notes on distributions, habitats and ecology, phenology, preliminary conservation assessments, etymology, vernacular names, uses, and specimens examined. An identification key to the species of the sect. Discostigma is presented.

## ﻿Materials and methods

The collected specimens were examined by consulting taxonomic literature (Anderson 1874; Kurz 1874, 1877; Pierre 1882, 1883; King 1890; Engler 1893; Vesque 1893; Pitard 1910; Ridley 1920, 1922; Gagnepain 1943; Backer and Bakhuizen van den Brink 1963; Maheshwari 1964; Chin 1973; Kochummen and Whitmore 1973; Whitmore 1973; Jones 1980; Singh 1993; Ngernsaengsaruay and Suddee 2022; Mohanan et al. 2023; Ngernsaengsaruay et al. 2024b), and by comparing with herbarium specimens housed in the following herbaria: AAU, BK, BKF, BM, C, CMUB, K, P, PSU, QBG, SING, and those included in the virtual herbarium databases of A (including GH), AAU, BM, CAL, E, G, K, L (including U), P, The Wallich Catalogue Online, US, W, and BO, MO, and NY (from GBIF, https://www.gbif.org/). All herbarium acronyms follow Thiers (2025, continuously updated). All specimens cited have been seen by the authors unless stated otherwise. The taxonomic history of the species was compiled using the literature and online databases (IPNI 2025; POWO 2025). The morphological characters, distributions, habitats and ecology, phenology, and uses were described from historic and newly collected herbarium specimens and the authors’ observations during fieldwork. The vernacular names were compiled from the specimens examined and the literature (e.g., Pooma and Suddee 2014; Ngernsaengsaruay and Suddee 2022; Ngernsaengsaruay et al. 2024b). Thailand’s floristic regions follow “Flora of Thailand” Vol. 4(3.3) (The Forest Herbarium, Department of National Parks, Wildlife and Plant Conservation 2023). The assessment of conservation status was performed following the IUCN Red List Categories and Criteria (IUCN Standards and Petitions Committee 2024), combined with GeoCAT analysis (Bachman et al. 2011) and field information.

## ﻿Results and discussion

### ﻿Taxonomic treatment

#### ﻿ Garcinia sect. Discostigma

Taxon classificationPlantaeMalpighialesClusiaceae

(Haask.) Hook. f., Gen. Pl. [Benth. & Hook. f.] 1: 174 (1862); M.Gaudeul, P. W.Sweeney & Munzinger, PhytoKeys 239: 90. 2024.

2A704319-C793-5B23-B689-3922CC323CF6

≡ Discostigma Hassk., Flora 25(2, Beibl.): 33. 1842. Type. Discostigma rostratum Hassk. ≡ Garcinia rostrata (Hassk.) Miq.

##### Description.

***Habit*** evergreen trees, dioecious; exudate yellow (in *Garcinia minutiflora*), pale yellow (in *G. santisukiana*), white, turning creamish white (in *G. merguensis*) or creamish white (in *G. rostrata*), sticky; branches decussate, horizontal or nearly horizontal; branchlets 4-angular, glabrous. ***Terminal bud*** concealed between the bases of the uppermost pair of petioles. ***Leaves*** decussate; lamina coriaceous or subcoriaceous, glabrous; secondary veins curving towards the margin and connected in distinct loops and united into an intramarginal vein, with interrupted long wavy lines (glandular wavy lines, also called exudate-containing canals) of differing lengths; petiole grooved above, with a small basal appendage clasping the branchlet. ***Inflorescences*** terminal, axillary, or on branchlets at leafless nodes (in axils of fallen leaves), in fascicles (clusters) of 2–10-flowered cymes, sometimes in a short thyrse of 5–12 flowers (in male inflorescences of *G. minutiflora*), or a solitary flower (in female flowers of *G. minutiflora* and *G. santisukiana*). ***Flowers*** unisexual, 4-merous; bracteoles 2 or 4, triangular or semi-orbicular; sepals and petals decussate; sepals semi-orbicular, broadly ovate, ovate, suborbicular, or orbicular; petals broadly elliptic, elliptic, orbicular, suborbicular, or obovate. ***Male flowers***: stamens numerous, united into 4 bundles, antepetalous (opposite the petals); anthers small, sessile to subsessile, 2-thecous; pistillode mushroom-shaped (fungiform). ***Female flowers***: staminodes present (in *G. santisukiana*) or absent (in *G. merguensis*, *G. rostrata*); pistil mushroom-shaped; ovary unlobed, bilocular, sometimes unilocular or four-locular (in *G. yunnanensis*) (Gaudeul et al. 2024); stigma sessile, unlobed, and smooth (Gaudeul et al. 2024) or weakly to shallowly lobed and papillate. ***Fruits*** berries with a sticky yellow (*G. minutiflora*, *G. santisukiana*) or white, turning creamish white (*G. merguensis*, *G. rostrata*) exudate secreted from cut fruits, subglobose, globose, ovoid, or broadly ellipsoid, with a smooth surface and unlobed, with coriaceous pericarp; persistent stigma discoid and shallowly concave (in *G. merguensis*, *G. rostrata*), flattened (in *G. santisukiana*), or convex (*G. minutiflora*); persistent sepals small. ***Seeds*** 1–2, reddish brown, dark brown, black, or brown mottled with pale brown, depressed subglobose, depressed globose, or compressed, one side flat, another side slightly convex (elliptic or oblong in outline).

Garcinia sect. Discostigma is characterised by its terminal or axillary, or on branchlets at leafless nodes, cymose inflorescences in fascicles of 2–10 flowers, sometimes in a short thyrse of 5–12 flowers, or a solitary flower; flowers with 4 sepals and 4 petals; male flowers with numerous stamens united into 4 bundles and with sessile to subsessile, 2-thecous anthers, with a pistillode; bilocular ovaries, sometimes unilocular or four-locular; weakly to shallowly lobed or unlobed and papillate or smooth stigmas; and fruits with a smooth surface and unlobed, with coriaceous pericarp, with a discoid persistent stigma, sometimes with a flattened or a convex persistent stigma, and with small persistent sepals. Distinguishing sectional morphological characters reported here were partly taken from Jones (1980) and Gaudeul et al. (2024).

According to Gaudeul et al. (2024), the unlobed and smooth stigmas are distinguishing sectional characters. However, from our examinations, all Thai species of Garcinia sect. Discostigma have weakly to shallowly lobed and papillate stigmas.

As stated by Gaudeul et al. (2024), the fruits of Garcinia sect. Discostigma are capped with a conspicuous discoid stigma. Furthermore, from the author’s observations, we found the persistent stigma in fruits of this section can be discoid and shallowly concave (in *G. merguensis*, *G. rostrata*), flattened (in *G. santisukiana*), or convex (in *G. minutiflora*).

A section of 60 species worldwide (Gaudeul et al. 2024), four species in Thailand [*i.e.*, *Garcinia merguensis* Wight, *G. minutiflora* Ridl., *G. rostrata* (Hassk.) Miq., and *G. santisukiana* Ngerns. & Suddee)]. Numbers of species in Garcinia sect. Discostigma recognised by Jones (1980) and Gaudeul et al. (2024) is shown in Table 1.

**Table 1.** Numbers of species in Garcinia section Discostigma recognised by Jones (1980), Gaudeul et al. (2024), and in this study.

Jones (1980) subsect. Discostigma	Gaudeu et al. (2024)	In this study
1. *Garcinia apetala* Pierre	1. *Garcinia apetala* Pierre	–
2. *Garcinia balansae* Pierre	2. *Garcinia balansae* Pierre	–
3. *Garcinia balica* Miq.	3. *Garcinia balica* Miq.	–
4. *Garcinia binnendijkii* Pierre	4. *Garcinia binnendijkii* Pierre	–
5. *Garcinia boerlagii* Pierre	5. *Garcinia boerlagii* Pierre	–
6. *Garcinia brevirostris* Scheff.	6. *Garcinia brevirostris* Scheff.	syn. nov. of *Garcinia rostrata* (Hassk.) Miq.
–	7. *Garcinia cadelliana* King	–
7. *Garcinia calophylla* Pierre	8. *Garcinia calophylla* Pierre	syn. nov. of *Garcinia rostrata* (Hassk.) Miq.
8. *Garcinia calophyllifolia* Ridl.	9. *Garcinia calophyllifolia* Ridl.	–
9. *Garcinia caudiculata* Ridl.	10. *Garcinia caudiculata* Ridl.	–
–	11. *Garcinia cordata* Merr.	–
10. *Garcinia cuneifolia* Pierre	12. *Garcinia cuneifolia* Pierre	–
11. *Garcinia cuspidata* King	13. *Garcinia cuspidata* King	–
12. *Garcinia daedalanthera* Pierre	* Garcinia daedalanthera* Pierre transferred to Garcinia section Hebradendron Planch. & Triana	–
13. *Garcinia diversifolia* King	14. *Garcinia diversifolia* King	–
14. *Garcinia dives* Pierre	15. *Garcinia dives* Pierre	–
15. *Garcinia dryobalanoides* Pierre	16. *Garcinia dryobalanoides* Pierre	–
–	17. *Garcinia enthaematoeides* Lauterb.	–
16. *Garcinia eugeniifolia* Wall. ex T. Anderson	–	= *Garcinia rostrata* (Hassk.) Miq.
17. *Garcinia fulva* Pierre	–	= *Garcinia merguensis* Wight
–	18. *Garcinia gitingensis* Elmer	= *Garcinia rostrata* (Hassk.) Miq.
18. *Garcinia grandifolia* (Choisy) Pierre	19. *Garcinia grandifolia* (Choisy) Pierre	–
19. *Garcinia hasskarlii* Pierre	20. *Garcinia hasskarlii* Pierre	–
–	21. *Garcinia havilandii* Stapf	–
20. *Garcinia holttumii* Ridl.	22. *Garcinia holttumii* Ridl.	–
21. *Garcinia hunsteinii* Lauterb.	23. *Garcinia hunsteinii* Lauterb.	–
–	24. *Garcinia jensenii* W. E. Cooper	–
22. *Garcinia keenania* Pierre	25. *Garcinia keenania* Pierre	–
–	26. *Garcinia kwangsiensis* Merr. ex F. N. Wei	–
23. *Garcinia lanceola* Ridl.	27. *Garcinia lanceola* Ridl.	–
–	28. *Garcinia lancilimba* C. Y. Wu ex Y. H. Li	–
24. *Garcinia lanessanii* Pierre	29. *Garcinia lanessanii* Pierre	–
25. *Garcinia linearis* Pierre	30. *Garcinia linearis* Pierre	–
26. *Garcinia linii* C. E. Chang	31. *Garcinia linii* C. E. Chang	–
27. *Garcinia luzoniensis* Merr.	32. *Garcinia luzoniensis* Merr.	–
28. *Garcinia memecyloides* Ridl.	33. *Garcinia memecyloides* Ridl.	–
29. *Garcinia merguensis* Wight	34. *Garcinia merguensis* Wight	1. *Garcinia merguensis* Wight
–	35. *Garcinia microphylla* Merr.	–
30. *Garcinia minimiflora* Ridl.	36. *Garcinia minimiflora* Ridl.	–
31. *Garcinia minutiflora* Ridl.	37. *Garcinia minutiflora* Ridl.	2. *Garcinia minutiflora* Ridl.
32. *Garcinia monantha* Ridl.	38. *Garcinia monantha* Ridl.	–
33. *Garcinia multiflora* Champ. ex Benth.	39. *Garcinia multiflora* Champ. ex Benth.	–
34. *Garcinia murtonii* Whitmore	40. *Garcinia murtonii* Whitmore	–
–	41. *Garcinia myrtifolia* A. C. Sm.	–
–	42. *Garcinia novoguineensis* Vesque	–
35. *Garcinia palawanensis* Elmer = *Garcinia dives* Pierre (POWO 2025)	–	–
36. *Garcinia picrorhiza* Miq.	43. *Garcinia picrorhiza* Miq.	–
37. *Garcinia rostrata* (Hassk.) Miq.	44. *Garcinia rostrata* (Hassk.) Miq.	3. *Garcinia rostrata* (Hassk.) Miq.
38. *Garcinia salakensis* Pierre	45. *Garcinia salakensis* Pierre	–
39. *Garcinia sampitana* Diels	46. *Garcinia sampitana* Diels	–
–	47. *Garcinia santisukiana* Ngerns. & Suddee	4. *Garcinia santisukiana* Ngerns. & Suddee
40. *Garcinia sarawhensis* Pierre	48. *Garcinia sarawhensis* Pierre	–
41. *Garcinia scaphopetala* B. L. Burtt	49. *Garcinia scaphopetala* B. L. Burtt	–
42. *Garcinia tauensis* Lauterb.	50. *Garcinia tauensis* Lauterb.	–
43. *Garcinia terpnophylla* Thwaites	51. *Garcinia terpnophylla* Thwaites	–
–	52. *Garcinia tetralata* C. Y. Wu ex Y. H. Li	–
44. *Garcinia travancorica* Bedd.	53. *Garcinia travancorica* Bedd.	–
45. *Garcinia treubii* Pierre	54. *Garcinia treubii* Pierre	–
46. *Garcinia umbellulata* Ridl.	–	–
47. *Garcinia umbonata* Lauterb.	55. *Garcinia umbonata* Lauterb.	–
–	56. *Garcinia versteegii* Lauterb.	–
48. *Garcinia vitiensis* (A. Gray) Seem.	57. *Garcinia vitiensis* (A. Gray) Seem.	–
49. *Garcinia warrenii* F. Muell.	–	–
50. *Garcinia wollastonii* Ridl.	58. *Garcinia wollastonii* Ridl.	–
–	59. *Garcinia yunnanensis* Hu	–
–	60. *Garcinia zichii* W. E. Cooper	–
51. *Garcinia* sp. A1 Whitmore	–	–
52. *Garcinia* sp. A2 Whitmore	–	–
53. *Garcinia* sp. A3 Whitmore	–	–

### ﻿A key to the species of Garcinia sect. Discostigma in Thailand

**Table:** 

1	Exudate creamish white or white, turning creamish white, secreted from cut stems and twigs; exudate white, secreted from cut fruits. Leaves lanceolate, elliptic, narrowly or broadly elliptic; apex tapering to a long blunt tip or acute; lamina without black gland dots; fresh leaves tough (not brittle) when crushed. Male inflorescences in fascicles of 3–10 flowers. Fruits with a discoid and shallowly concave persistent stigma; fruiting stalk longer (3–8 mm long)	**2**
–	Exudate pale yellow or yellow, secreted from cut stems and twigs; exudate yellow, secreted from cut fruits. Leaves obovate or elliptic; apex obtuse, acute, or retuse (not tapering to a long blunt tip); lamina with scattered black gland dots; fresh leaves brittle when crushed. Male inflorescences in short thyrses of 5–12 flowers or in fascicles of 3–5 flowers. Fruits with a convex or a flattened persistent stigma; fruiting stalk shorter (1.5–3 mm long)	**3**
2	Leaf apex acute or tapering to a long blunt tip, 0.6–1 cm long; angle between midrib and secondary veins 45°–55°; intersecondary veins 2.5–3.5 mm apart from secondary veins; young leaves reddish brown. Male flowers larger (0.7–1.2 cm diam.)	**1. *Garcinia merguensis***
–	Leaf apex tapering to a long blunt tip, (0.7–)1–2 cm long; angle between midrib and secondary veins 65°–80°; intersecondary veins 1.5–2 mm apart from secondary veins; young leaves red or pale greenish red. Male flowers smaller (5–6.5 mm diam.)	**3. *Garcinia rostrata***
3	Leaves apex obtuse or retuse; secondary veins 5–9 on each side, with many scattered black gland dots below. Male inflorescences in short thyrses of 5–12 flowers (up to 2 cm long). Flowers smaller (1.8–2 mm diam.). Fruits with a convex persistent stigma. Limestone species	**2. *Garcinia minutiflora***
–	Leaves apex acute, sometimes retuse; secondary veins 9–14 on each side, with scattered black gland dots on both surfaces. Male inflorescences in fascicles of 3–5 flowers. Flowers larger (4–7 mm diam.). Fruits with a flattened persistent stigma. Non-limestone species	**4. *Garcinia santisukiana***

#### ﻿1. Garcinia merguensis

Taxon classificationPlantaeMalpighialesClusiaceae

Wight, Ill. Ind. Bot. [Madras] 1(pts. 1–8): 124. 1838 et Icon. Pl. Ind. Orient. 1(10): 6. t. 116. 1839

66DAD306-AAEF-5A18-AFE8-6F54D4F94553

Figs 1 , 2 , 3 , 4

Garcinia merguensis Wight, Ill. Ind. Bot. [Madras] 1(pts. 1–8): 124. 1838 et Icon. Pl. Ind. Orient. 1(10): 6. t. 116. 1839; T. Anderson in Hook. f., Fl. Brit. India 1(2): 267. 1874; Kurz, J. Asiat. Soc. Bengal, Pt. 2, Nat. Hist. 43(2): 87. 1874 et Forest Fl. Burma 1: 89. 1877; Pierre, Fl. Forest. Cochinch. 1(5): 6. t. 91D. 1883; King, J. Asiat. Soc. Bengal, Pt. 2, Nat. Hist. 59(2): 150. 1890; Engl. in Engl. & Prantl, Die Naturlichen Pflanzenfamilien 3(6): 236. 1893; Vesque in A. DC. & C. DC., Monogr. Phan. 8: 341. 1893; Brandis, Indian Trees: 51. 1906; Pit. in Lecomte et al., Fl. Indo-Chine 1(4): 299. 1910; Ridl., Fl. Malay Penins. 1: 169. fig. 19. 1922; Craib, Fl. Siam. 1(1): 116. 1925; Gagnep. in Humbert & Gagnep., Fl. Indo-Chine Suppl.: 258. 1943; Maheshw., Bull. Bot. Surv. India 6: 118. 1964; Corner & Watan., Ill. Guide Trop. Pl.: t. 191. 1969; Kochummen & Whitmore, Gard. Bull. Singapore 26(2): 278. 1973; Whitmore in Whitmore, Tree Fl. Malaya 2: 215. 1973; S. W. Jones, Morphology and Major Taxonomy of Garcinia (Guttiferae), Ph.D. Thesis (unpublished): 368. fig. 7/12. 1980; P. H. Hô, Câyco Vietnam 1: 565. fig. 1562. 1991; N. P. Singh in B. D. Sharma & Sanjappa, Fl. Ind. 3: 118. 1993; M. Turner, Gard. Bull. Singapore 47(1): 262. 1995; S. Gardner, P. Sidisunthorn & V. Anusarnsunthorn, Field Guide Forest Trees of N. Thailand: 51. fig. 55. 2000; S. Gardner, P. Sidisunthorn & Chayam., Forest Trees S. Thailand 1: 356. fig. 547. 2015; N. Mohanan et al. Rheedea 33(3): 152. 2023.
≡ Discostigma merguense (Wight) Planch. & Triana, Ann. Sci. Nat., Bot., sér. 4, 14: 363. 1860. Type. lectotype [designated by Maheshwari (1964)], Myanmar, Mergui, fl. & young fr., 1860, *W. Griffith 96*, transcription error as *W. Griffith 97*, K! [K000677610] (Fig. 3A).
= Garcinia merguensis Wight var. truncata Pierre, Fl. Forest. Cochinch. 1(5): t. 68. 1883; Vesque in A. DC. & C. DC., Monogr. Phan. 8: 343. 1893; Pit. in Lecomte et al., Fl. Indo-Chine 1(4): 300. 1910 (as var. *typica* Pierre in the type). Type. lectotype (designated here), Vietnam, ad Cay Cong in prov. Tayninh gallicae austro Cochinchinae, ♂ fl., Jun 1866, *J. B. L. Pierre 3630*, P digital image! [P05061522] (Fig. 3B); isolectotypes: L digital image! [U1215669, U1215671]; P! [P05061445, P05061521].
= Garcinia merguensis Wight var. pyramidata Pierre, Fl. Forest. Cochinch. 1(5): t. 69. 1883; Vesque, Epharmosis 2: 15. t. 96, 97. 1889 et in A. DC. & C. DC., Monogr. Phan. 8: 343. 1893; Pit. in Lecomte et al., Fl. Indo-Chine 1(4): 300. 1910. Type. lectotype (designated here), Cambodia, ad montem Cam Chay in prov. Kampot, ♂ fl., May 1874, *J. B. L. Pierre 3638*, P! [P05062835] (Fig. 4A); isolectotypes: L digital image! [U1215668], P! [P05062839, P05062843, P05062844].
= Garcinia fulva Pierre, Fl. Forest. Cochinch. 1(5): 6. t. 92B. 1883; Engl. in Engl. & Prantl, Die Naturlichen Pflanzenfamilien 3(6): 236. fig. 111A. 1893; Vesque in A. DC. & C. DC., Monogr. Phan. 8: 361. 1893. Type. lectotype (designated here), Peninsular Malaysia, Malacca, fl. & fr., s.d., *W. S. Kurz & A. C. Maingay s.n.*, P! [P04701647].
= Garcinia lanceolata Ridl., Fl. Malay Penins. 1: 170. 1922; Craib, Fl. Siam. 1(1): 116. 1925; Kochummen & Whitmore, Gard. Bull. Singapore 26(2): 278. 1973. Type. lectotype (designated here), Malaysia, Langkawi, Pulau Terutau, Telok Udang, fl., Sep 1914, M. Haniff 1073, K! [K000677649] (Fig. 4B); isolectotype: SING! [SING0063109].

##### Description.

***Habit*** trees, 3–15(–20) m tall, 15–80(–120) cm GBH; exudate white, turning creamish white, sticky; branchlets green, 4-angular, glabrous. ***Bark*** brown or dark brown, slightly rough or scaly; inner bark pale brown. ***Leaves*** lanceolate, elliptic, narrowly or broadly elliptic, 6.4–13.7 × 2.8–6.2 cm, apex acute or tapering to a long blunt tip, 0.6–1 cm long, base cuneate, margin entire or repand, coriaceous, dark green above, paler below, glabrous on both surfaces, midrib slightly raised above, raised below, secondary veins 13–18 on each side, 4–7 mm apart from each other, departing from the midrib at an angle of 45°–55°, curving towards the margin and connected in distinct loops and united into an intramarginal vein, visible on both surfaces, with intersecondary veins, 2.5–3.5 mm apart from secondary veins, veinlets reticulate, faint above, visible below, interrupted long wavy lines present, of differing lengths, nearly parallel to the midrib, running across the secondary veins to the apex, visible below; petiole 5–8.5 mm long, 1–1.5 mm diam., grooved above, glabrous, with a small basal appendage clasping the branchlet; fresh leaves tough (not brittle) when crushed; young leaves reddish brown, turning pale green, glossy. ***Inflorescences*** terminal or axillary, in fascicles of 2–8-flowered cymes or solitary (number of flowers per inflorescence of male inflorescences more than female inflorescences). ***Flowers*** unisexual; bracteoles 2, opposite; sepals and petals decussate, concave, glabrous. ***Flower buds*** green, subglobose or globose, 2–4 mm diam. ***Male flowers*** in fascicles of 3–8 flowers, 0.7–1.2 cm diam.; bracteoles triangular, 1–1.7 × 0.9–1.5 mm, apex acute; pedicel green, 2–6.5 mm long, 0.6–1.5 mm diam., glabrous; sepals 4, pale green, the outer pair smaller than the inner pair, the outer pair semi-orbicular, 1–2 × 1–2.1 mm, apex obtuse, the inner pair broadly ovate, 2.3–3.5 × 2–5 mm, apex obtuse; petals 4, pale yellow or yellow, broadly elliptic or orbicular, 3–5.7 × 3.2–5.7 mm, subequal, apex obtuse or rounded; stamens numerous, united into 4 bundles, each bundle 1.7–4 × 1–4 mm; filaments very short; anthers small; pistillode mushroom-shaped, 2.5–4.5 mm long; sterile stigma sessile, convex, 2.8–3.7 mm diam., papillate. ***Female flowers*** in fascicles of 2–3 flowers or solitary, 4.5–8.5 mm diam.; bracteoles triangular, 0.5–1.1 × 0.5–1 mm, apex acute; pedicel green, 2.5–6 mm long, 0.3–0.5 mm diam., glabrous; sepals 4, pale green, the outer pair smaller than the inner pair, the outer pair semi-orbicular, 1–2.2 × 1–2.5 mm, apex obtuse, the inner pair broadly ovate or orbicular, 2.5–4.7 × 2.2–5.5 mm, apex obtuse; petals 4, pale yellow or yellow, broadly elliptic, elliptic, or orbicular, 3–6 × 3.2–6 mm, subequal, apex rounded or obtuse; staminodes absent; pistil mushroom-shaped; ovary pale green, subglobose, 1.5–3.5 × 2–3.5 mm; stigma bright red or pale yellow, sessile, convex, 2.3–5 mm diam., unlobed, papillate. ***Fruits*** berries, subglobose or globose, 1.7–2.3 × 1.7–2.4 cm, green or dark green, turning yellow when ripe, smooth and glabrous, pericarp coriaceous, c. 2.5–5 mm thick, cut fruits with a sticky white exudate, with persistent sepals; persistent stigma blackish brown or dark brown, discoid and shallowly concave, 4–5.5 mm diam., unlobed, papillate; fruiting stalk 5–8 mm long, 1.2–1.5 mm diam. ***Seeds*** 1–2, reddish brown or dark brown, depressed subglobose or depressed globose, 1.4–1.7 × 1.2–1.7 cm, with a thin fleshy pulp.

##### Distribution.

India (Assam, Andaman and Nicobar Islands), Bangladesh, Myanmar (Tenasserim, Mergui Archipelago), Vietnam, Laos, Cambodia, Thailand, Peninsular Malaysia [Kedah (including Langkawi Island), Penang, Perak, Kelantan, Pahang, Malacca], Singapore, Indonesia [Sumatra, reported by Jabit et al. (2009)], Borneo [Malaysia (Sarawak), Indonesia (Kalimantan)]. (Fig. 5).

##### Distribution in Thailand.

It is found in all floristic regions. **Northern**: Chiang Mai, Chiang Rai, Phayao, Nan, Lamphun, Lampang, Tak, Phitsanulok; **North-Eastern**: Loei; **Eastern**: Nakhon Ratchasima; **South-Western**: Uthai Thani, Kanchanaburi, Ratchaburi, Prachuap Khiri Khan; **Central**: Nakhon Nayok; **South-Eastern**: Chon Buri, Chanthaburi, Trat; **Peninsular**: Ranong, Surat Thani, Phangnga, Phuket, Krabi, Nakhon Si Thammarat, Phatthalung, Trang, Satun, Songkhla, Pattani, Yala, Narathiwat. (Fig. 5).

##### Habitat and ecology.

This species is found in a wide variety of habitats, including tropical evergreen rain forests, dry evergreen forests, littoral dry evergreen forests, beach forests, lower montane rain forests, and secondary forests, often along streams, at elevations of 0–1,500 m a.m.s.l.

##### Phenology.

Flowering and fruiting more than once, nearly throughout the year, with a peak from February to May.

##### Conservation status.

*Garcinia merguensis* is widely distributed from Assam to Sumatra and Borneo. It is known from many localities and has a large Extent of Occurrence (EOO) of 2,704,316.60 km^2^ and an Area of Occupancy (AOO) of 180 km^2^. In Thailand, this species is known to be naturally distributed throughout the seven floristic regions and has an EOO of 638,851.52 km^2^ and an AOO of 100 km^2^. Because of its wide distribution and the number of localities, it does not face any threat of extinction. We therefore suggest the conservation assessment here as Least Concern (LC) in agreement with de Kok (2024a).

##### Etymology.

The specific epithet of *Garcinia merguensis* was named after the Mergui Archipelago, southern Myanmar, where William Griffith (1810–1845), a British colonial physician and botanist, collected the type specimen.

##### Vernacular names.

Kanuan (กะนวล) (Prachuap Khiri Khan, from the specimen *Winit 621*); Kraduk kai (กระดูกไก่) (Nakhon Si Thammarat, from the specimen *Snan 922*); Kraduk ngu (กระดูกงู) (Ranong, from the specimen *Pochanart 426*); Kraduk nok (กระดูกนก) (Trat, from the specimen *Bunnak 433*); Khanom pang (ขนมปัง) (Chanthaburi, from the specimen *Noe 61*); Khi phueng (Surat Thani, from the specimen *Sanan 399*); Sa-pae (ซาแป) (Malay-Narathiwat, reported by Pooma and Suddee 2014); Duk khang (ดูกค่าง) (Trang, from the specimen *S. Phusomsaeng 217*); Dok khang (โดกค่าง) (Trang, from the specimen *B. Sangkhachand 1883*); **Nuan** (**นวล**) (Trang, from the specimen *C. Bunnab 65*); Nuan paeng (นวลแป้ง) (Nakhon Si Thammarat, from the specimen *Rabil 200*); Bun yong (บุญยง) (Lampang, from the specimen *Bunnak 25*); Prik (ปริก) (Trang, from the specimen *C. Niyomdham & W. Ueachirakan 1779*); Muang sai (มวงทราย) (Trang, from the specimen *B. Sangkhachand 1901*); Muang nok (มวงนก) (Ranong, from the specimen *A. F. G. Kerr 16710*); Yang khao (ยางเขา) (Trat, from the specimen *Put 2961*); Yai pluak (ยายปลวก) (Trang, from the specimen *S. Boonkerd 42*); Wi lai (วิไล) (Trang, from the specimen *V. Chamchumroon et al. 1/3*); Lulai (Malay, Ridley 1922).

##### Uses.

The fruits of *Garcinia merguensis* are a wildlife food source. In West Sumatra, Indonesia, the fruits of this species are eaten by local people, and the bark is used to produce a yellow dye, while the leaves are used in folk medicine for the treatment of edema (Jabit et al. 2009).

##### Lectotypifications.

In the original publication of Garcinia merguensis var. truncata by Pierre (1883: t. 68), only one collection is cited, *Herb. Pierre 3630*. However, from our examination, we found this collection has two localities and dates, and it can be separated as two groups: Group A at L [U1215669, U1215671], P [P05061445, P05061521, P05061522] collected from ad Cay Cong in prov. Tây Ninh gallicae austro Cochinchinae in June 1866 and Group B at P [P05061516, P05061517, P05061523] collected from ad Noc in prov. Baria gallicae austro Cochinchinae in June 1869. Following Art. 9.6 of the ICN (Turland et al. 2018), these are syntypes. The P [P05061522] specimen of Group A is better preserved and more complete than the others, and hence is chosen here as the lectotype, following Arts. 9.3 and 9.12 of the ICN (Turland et al. 2018).

Garcinia merguensis var. pyramidata was named by Pierre (1883: t. 69), who cited three sets of material: *Herb. Pierre 613, 615*, and *3638*. He did not choose a holotype, and following Art. 9.6 of the ICN (Turland et al. 2018), they constitute syntypes. We located the materials (1) *J. B. L. Pierre 613* collected from montibus Knang Repoeu, prov. Tpong, with two dates: Group A at P [P05062842] on 27 May 1870 and Group B at K [without barcode], P [P05062836, P05062841] in June 1870; (2) *J. B. L. Pierre 615* collected from montibus Knang Repoeu, prov. Tpong, with two dates: Group A at K [without barcode], P [P05062847, P05062850] in May 1870 and Group B at K [without barcode], L [U1215670], P [P05061550, P05062845, P05062846, P05062851] in June 1870; and (3) *J. B. L. Pierre 3638* collected from ad montem Cam Chay prov. Kampot at L [U1215668], [P05062835, P05062839, P05062843, P05062844] on May 1874. From the third set of materials, the P [P05062835] specimen is in the best condition and clearly shows the diagnostic characters for the species and is selected here as the lectotype, following Arts. 9.3 and 9.12 of the ICN (Turland et al. 2018).

*Garcinia fulva* was described by Pierre (1883: 6. t. 92B), based on the specimen *Maingay & Kurz s.n.* collected from Malacca. He did not designate a holotype nor did he mention the name of the herbarium where the specimen was housed. However, we found the specimen *W. S. Kurz & A. C. Maingay s.n.* at P [P04701647], and following Art. 9.6 of the ICN (Turland et al. 2018), it constitutes a syntype. Therefore, the P [P04701647] specimen is selected here as the lectotype, following Arts. 9.3 and 9.12 of the ICN (Turland et al. 2018).

*Garcinia lanceolata* was named by Ridley (1922: 170), who cited the type locality and the collector’s name “Langkawi, Pulau Terutau at Telok Hudang (*Haniff*)”. He did not choose a holotype nor did he mention the collector number and the name of the herbaria where the material was housed. However, we located the collection *M. Haniff 1073* from Langkawi (originally “Pulau Terutau, Telok Udang” on the label) at K [K000677649] and SING [SING0063109], and following Art. 9.6 of the ICN (Turland et al. 2018), they constitute syntypes. Henry Nicholas Ridley (1855–1956) was a director of Botanic Gardens Singapore (1888–1912); then after his retirement from SING, he was based at K (Stafleu and Cowan 1983). Therefore, the K [K000677649] specimen is designated here as the lectotype, following Arts. 9.3 and 9.12 of the ICN (Turland et al. 2018).

##### Notes.

*Garcinia merguensis* was described by Wight (1838: 124), who cited the material *W. Griffith 97* collected from Mergui. He did not select a holotype, nor did he mention the name of the herbarium where the material was housed. Maheshwari (1964) designated the same material at K [without barcode] as the lectotype. However, from our examination, the specimen number appears to be *W. Griffith 96* (in Herb. Mergui) at K [K000677610], so perhaps there has been a transcription error as *W. Griffith 97* in the original description and Maheshwari (1964).

*Garcinia merguensis* has a white exudate that turns creamish white, secreted from cut stems and twigs, while *G. rostrata* has exudate a creamish white exudate secreted from cut stems and twigs.

##### Additional specimens examined.

**Thailand.** • **Northern**: Chiang Mai [Doi Suthep, ♀ fl. & fr., 24 Apr 1910, *A. F. G. Kerr 1135* (BM, K); • ibid., ♂ fl., 24 Apr 1910, *A. F. G. Kerr 1139* (BM, K, P [P05061525]); • ibid., young fr., 20 Nov 1910, *A. F. G. Kerr 1156A* (BM); • ibid., fl., 14 Apr 1921, *A. F. G. Kerr s.n.* (BM); • ibid., fr., 15 Apr 1921, *A. F. G. Kerr 5257* (AAU, BK, BM, K); • ibid., fl., 28 Apr 1958 (as *Garcinia* sp.), *T. Sørensen et al. 3108* (BKF, C); • ibid., 24 Jun 1958 (as *Garcinia* sp.), *T. Sørensen et al. 3712* (BKF, C); • ibid., 6 Feb 1959 (as *Garcinia* sp.), *T. Sørensen et al. 6855* (BKF, C); • ibid., fr., 6 Feb 1988, *J. F. Maxwell 88-132* (L [L2416490]); • ibid., ♂ fl., 28 Apr 1988, *J. F. Maxwell 88-545* (AAU, BKF, L [L2416498]); • Doi Suthep-Pui National Park, ♂ fl., 2 May 1990, *J. F. Maxwell 26* (CMUB); • ibid., ♂ fl., 13 May 1993, *J. F. Maxwell 93-420* (CMUB, L [L2416504, L2416505]); • ibid., fr., 5 May 1997, *J. Panyadit s324b1* (CMUB); • ibid., fr., 5 May 1998 (as *G. euginiifolia*), *C. Kuarak s324b2* (CMUB); • Doi Suthep, fl., 15 Jun 2012 (as *Garcinia* sp.), *M. Norsaengsri et al. 9417* (QBG); • Pha Mon, Chom Thong District, ♂ fl., 20 Feb 1939, *Somkhid 301* (BKF); • Huai Nam Dang National Park, Mae Taeng District, ♂ fl., 27 May 1977 (as *Garcinia* sp.), *T. Santisuk 1051A* (BKF, PSU); • ibid., ♀ fl., 27 May 1977 (as *Garcinia* sp., *G. rostrata*), *T. Santisuk 1051B* (AAU, BKF, PSU); • Doi Chiang Dao, young fr., 2 Dec 1984 (as *Garcinia* sp.), *W. Nanakorn 1033* (AAU); • Ban Pa Pae, Pa Pae Subdistrict, Mae Taeng District, fr., 5 Nov 1990, *J. F. Maxwell 90-1208* (AAU, L [L3806734]); • Botanic Garden, Mae Rim District, fr., 28 Feb 1994, *BGO. Staff 449 (434)* (QBG); • Ban Mae Kampong, Mae On District, ♂ fl., 22 Apr 1999 (as *Garcinia* sp.), *P. Kumphet et al. WP424* (QBG); • Ban Na Yao, Piang Luang Subdistrict, Wiang Haeng District, fl., 14 May 2001, *M. Norsaengsri 1533* (CMUB, QBG); • Doi Inthanon National Park, Mae Chaem District, fr., 24 May 2023, *C. Ngernsaengsaruay & T. Kaewgrajang G66-24052023* (BKF); • Huai Hom, Ban Chan Subdistrict, Mae Chaem District, fr., 11 Dec 2007 (as *Garcinia* sp.), *N. Tanaka HN8415* (QBG)]; • Chiang Rai [Doi Nang Kaeo, fr., 11 Mar 1912, *A. F. G. Kerr 2526* (AAU, BM, E [E00839791], K); • Khun Chae National Park, Wiang Pa Pao District, ♂ fl., 6 Apr 1998 (as *Garcinia* sp., *G. speciosa*), *J. F. Maxwell 98-406* (BKF, CMUB)]; • Phayao [Champathong Waterfall, Doi Luang National Park, Mueang Phayao District, ♂ fl., 27 May 1997 (as *Garcinia* sp.), *S. Gardner & P. Sidisunthorn 2152* (A [A00466353], CMUB, L [L3806370, L3813194])]; • Nan [Ban Tiu, fr., 2 Mar 1921, *A. F. G. Kerr 4976* (AAU, BK, BM, K); • Doi Phu Kha National Park, fr., 3 Sep 1938 (as *Garcinia* sp.), *Somkhid 215* (BKF); • ibid., fr., 22 Sep 1996 (as *Garcinia* sp.), *R. Pooma 1375* (CMUB11821); • ibid., fr., 8 Apr 1999 (as *G. cowa*), *P. Srisanga & S. Wathhana 618* (BKF, QBG); • ibid., ♂ fl., 1 Sep 2000, *P. Srisanga 1542* (QBG)]; • Lamphun [Mae Li, fr., 23 Apr 1915, *Winit 101* (BM, K); • Doi Khun Tan National Park, fl., 29 Apr 1994, *J. F. Maxwell 94-560* (BKF, CMUB, L [L3810671])]; • Lampang [Ban Pa Miang, Chae Son Subdistrict, fr., 20 Jan 1914, *A. F. G. Kerr 3121* (BM, E [E00160908], K); • Mae Yom, ♂ fl., 29 May 1927, *Winit 1827* (BK, BKF, K); • Ngao District, ♂ fl., 2 May 1954 (as *G. rostrata*), *Bunnak 25* (BKF); • Khun Tan, fr., 6 Mar 1998 (as Clusiaceae), BGO. Staff 10709 (QBG)]; • Tak [Huai Krasa, 90 km South of Tak, fr., 20 Mar 1968, *B. Hansen & T. Smitinand 12998* (BKF, C)]; • Phitsanulok [Water Wheel, Phu Hin Rong Kla National Park, fr., 14 Oct 1998 (as *Garcinia* sp.), *P. Suksathan 1314* (QBG); • Chat Trakan, fr., 22 Jan 2009, *C. Maknoi 3027* (QBG)]; **North-Eastern**: • Loei [Phu Kradueng, fl., 11 Sep 1954 (as *Garcinia* sp.), *T. Smitinand 1912* (BKF); Phu Kradueng, fr., 10 Dec 1974 (as *Garcinia* sp.), *T. Smitinand 12014* (BKF); • Phu Suan Sai National Park, Na Haeo District, fr., 15 May 2008 (as *Garcinia* sp.), *C. Maknoi & P. Srisanga 2297* (BKF, QBG); • ibid., fr., 11 Mar 2008 (as *Garcinia* sp.), *C. Maknoi 2051* (BKF); ibid., fr., 3 Sep 2008 (as *Garcinia* sp.), *C. Maknoi 2789* (BKF, QBG); Route to Tat Pha Waterfall, Na Haeo District, fl., 11 Jul 2008 (as *Garcinia* sp.), *C. Maknoi 2612* (BKF, QBG); • Huai Baeng Forest Protection Station, Phu Luang Wildlife Sanctuary, fr., 10 Mar 2009 (as *Garcinia* sp.), *T. Wongprasert 093-22* (BKF); • Huai Nam Baeng, Phu Luang Wildlife Sanctuary, sterile, 15 Jun 1994 (as *Garcinia* sp.), *T. Santisuk et al. s.n.* (BKF)]; **Eastern**: • Nakhon Ratchasima [Wang Nam Khiao District, ♂ fl., 20 Apr 1967, *D. Prapat 1* (BKF); • Sakaerat Environmental Research Station, Dec 1987 (as *Garcinia* sp.), *T. Smitinand 898* (BKF)]; • Ubon Ratchathani [Dong Fa Huan, fr., 24 Jun 2004 (as *Garcinia* sp.), cultivated, *P. Puudjaa 1330* (BKF)]; **South-Western**: • Uthai Thani [Huai Kha Khaeng Wildlife Sanctuary, Ban Rai District, fr., 20 Feb 1970 (as *Garcinia* sp.), *C. F. van Beusekom & T. Santisuk 2873* (AAU, BKF, C, P [P05061709])]; • Kanchanaburi [Thai-Myanmar border, Sangkhla Buri District, ♂ fl., 11 May 1946, *Kasin 367* (K, L [L2416513], P [P05061545], SING); • Khao Ngi Yai, E. of Sangkhla Buri District, ♀ fl. & fr. 5 Apr 1968 (as *Garcinia* sp.), *C. F. van Beusekom & C. Phengkhlai 361* (BKF 47189, AAU, K, P [P05062041]); • W. of Si Sawat District, ♂ fl., 27 Jun 1974, *K. Larsen & S. S. Larsen 33861* (AAU, E [E00160909], K, P [P05061539]); • Kroeng Krawia Waterfall, Thong Pha Phum District, fr., 24 Feb 1981, B. Thanee 326 (BKF073555); • North of Ti Nuai Forest Protection Unit, Thung Yai Naresuan Wildlife Sanctuary, fl., 2 May 1992 (as *Garcinia* sp.), *T. Santisuk et al. s.n.* (BKF115358); • Thong Pha Phum District, ♂ fl. (spirit collection), 3 May 2004, *C. Ngernsaengsaruay G61-03052004* (BKF); • Locality unspecified, fl., 4 May 1992 (as *Garcinia* sp.), *T. Santisuk et al. s.n.* (BKF105387); • Wat Phu Ye, Sangkhla Buri District, fr., 17 May 2018 (as *Garcinia* sp.), *N. Muangyen 3264* (QBG)]; • Ratchaburi [Thai-Myanmar border, between Tuang Province and Chom Bueng District, fl., 25 Mar 1975 (as *Garcinia* sp.), *J. F. Maxwell 75-324* (BK)]; • Prachuap Khiri Khan [Huai Di Buk, fl., 16 Jul 1921, *Winit 621* (BK, BKF, K); • Pa La U Noi, Kaeng Krachan National Park, fl., 16 Aug 2002 (as *Garcinia* sp.), *D. J. Middleton et al. 1128* (A [A00466328], BKF, CMUB, K); Huai Yang National Park, Thap Sakae District, fr., 6 Jan 2004 (as *Garcinia* sp.), *D. J. Middleton et al. 2522* (A [A00466331], BKF)]; **Central**: • Nakhon Nayok [Mueang Nakhon Nayok District, fl., 1 May 2002, *P. Charoenchai & S. Phomphuang 245* (BK, CMUB)]; **South-Eastern**: • Chon Buri [Ban Dan, Sriracha District, fl., 5 Mar 1920, *A. F. G. Kerr 4043* (BM)]; • Chanthaburi [Khao Khitchakut, ♂ fl., 14 Apr 1925, *Noe 61* (AAU, BK, BM, K, L [L2416515]); • Khao Khitchakut National Park, fl., 30 Mar 2008, *P. Phonsena 5912* (BKF); • Makham District, ♂ fl., 9 Apr 1959 (as *G. rostrata*), *T. Smitinand 5775* (BKF); • Khao Soi Dao Wildlife Sanctuary, sterile, 19 May 2013, S. *Tagane et al. T1720* (BKF); • Khlong Mayom, s.d., *J. Schmidt 613* (C)]; • Trat [Khao Saming, fr., 31 Mar 1925, *Noe 46* (AAU, BK, BM, K); • Khao Kuap, young fr., 27 Dec 1929, *A. F. G. Kerr 17845* (AAU, BK, BM, C, E [E00839786], K, L [L2416514]); • ibid., fr., 23 May 1930, *Put 2961* (AAU, BK, K); • Khlong Phrao, Laem Ngob, Ko Chang, fl., 2 May 1955 (as *G. rostrata*), *Bunnak 433* (BKF); • Khlong Munse, Ko Chang, 3 Apr 1959 (as *Garcinia* sp.), *T. Sørensen et al. 7133* (C); Khlong Nonsi, Ko Chang, ♂ fl., 3 Apr 1959 (as G. cf. rostrata), *T. Smitinand 5649* (BKF); • Ko Chang, ♂ fl., 11 Mar 1970 (as *Garcinia* sp.), *C. F. van Beusekom & T. Santisuk 3176* (AAU, BKF, C, K, P [P05062051]); • Ao Ong Kang, Ko Chang, ♀ fl., 7 May 1974 (as *Garcinia* sp., *G. cowa*), *R. Geesink et al. 6589* (AAU, BKF, C, K, P [P05061698]); • Khlong Takhian, Ko Chang, ♀ fl., 7 May 1974, *J. F. Maxwell 74-394* (AAU, BK); • Ko Chang, ♂ fl., 9 May 1974, *J. F. Maxwell 74-426* (AAU, BK); • Khlong Phlu Waterfall, Ko Chang, fl., 29 Mar 2000 (as *G. euginiifolia*), *T. Wongprasert s.n.* (BKF163961); Khlong Makok, Ko Chang, fl., 24 Mar 2001 (as *Garcinia* sp.), *T. Wongprasert 013-04* (BKF), *013-08* (BKF); • ibid., fl., 24 Mar 2001 (as *Garcinia* sp.), *K. Chayamarit et al. 2908* (BKF); • Ko Kut, fr., 22 Oct 2000 (*G. acuminata*), *C. Phengklai et al. 13115* (BKF); • ibid., ♂ fl., 22 Oct 2000, *C. Phengklai et al. 13116* (BKF); • Khlong Chao-Ao Phrao, Ko Kut, fl., 7 Apr 2002 (as *G. acuminata*), *C. Phengklai et al. 13359* (BKF); • ibid., fl., 7 Apr 2002 (*G. bancana*), *C. Phengklai et al. 13448* (BKF); • ibid., 15 Oct 2022, *C. Ngernsaengsaruay* personal observation; Mai Rut Subdistrict, Khlong Yai District, 19 Jul 2024, *C. Ngernsaengsaruay* personal observation]; **Peninsular**: • Ranong [Kapoe District, fr., 18 Jan 1929, *A. F. G. Kerr 16710* (BK, BM, E [E00839790], K); • Hua Sing, Kra Buri District, fl., 18 Apr 1967 (as *Garcinia* sp.), *S Sutheesorn 2291* (BK); • Hot Spring, fr., 22 Jan 1969 (as *Garcinia* sp.), *T. Smitinand & R. Schaller 10633* (BK); Khlong Nakha, fr., 25 Apr 1973 (as *Garcinia* sp.), *R. Geesink & T. Santisuk 4886* (AAU, BKF, C, K, P [P05062054]); • ibid., fl., 25 Apr 1973 (as *Garcinia* sp.), *R. Geesink & T. Santisuk 4908* (AAU, BKF, C, P [P05062009]); • ibid., fr., 22 Jun 1974 (as *Garcinia* sp.), *R. Geesink et al. 7401* (AAU, BKF, K, P [P05061702]); • ibid., fr., 9 Dec 1976 (as *Garcinia* sp.), *T. Santisuk 799* (BKF, PSU); • Locality unspecified, young fr., 8 Aug 1973, *Pochanart 415* (BKF); • Locality unspecified, young fr., 12 Aug 1973, *Pochanart 426* (BKF); Bang Ben, Kapoe District, ♂ fl., 25 Dec 1976, *T. Santisuk 847* (BKF, PSU); • Kapoe District, fl., 23 Aug 1977 (as *Garcinia* sp.), *T. Santisuk 1276* (BKF); • ibid., fr., 23 Aug 1977, *T. Santisuk 1277* (BKF, PSU); • Big Kam Island, Laem Son National Park, Kapoe District, ♂ fl., 1 Dec 1996 (as *G. euginiifolia*), *J. F. Maxwell 96-1587* (CMUB); • Ngao Waterfall, young fr., 8 Dec 1979, *T. Shimizu et al. T-26567* (BKF); • ibid., young fr., 9 Sep 1984 (as *Garcinia* sp.), *W. Nanakorn 701* (BKF); • Namtok Ngao National Park, fl., 19 Jan 2005 (as *Garcinia* sp.), *P. Sidisunthorn & P. Tippayasri ST1355* (K); Ban Bang Man, fl., 25 Apr 2005 (as *Garcinia* sp.), *R. Pooma et al. 5249* (AAU, BKF); • Ko Phayam, fr., 24 Jan 2008 (as *Garcinia* sp.), *B. Sonsupab s.n.* (BK)]; • Surat Thani [Thung Luang, fr., 1 Apr 1927, *A. F. G. Kerr 12517* (AAU, BK, BM, E [E00839788], K); • Ban Na San District, young fr., 14 Aug 1955, *Sanan 399* (BKF); • Khlong Sok, young fr., 12 Dec 1975, *D. Prapat 153* (AAU, BKF, C, K, L [L2416500], P [P05062011]); • Locality unspecified, fr., 17 Apr 1977, *C. Phengklai et al. 3874* (BKF, PSU); • Trail behind Khao Sok Ranger Station, Rajjaprabha Dam, young fr., 20 Feb 2001 (as *Syzygium* sp.), *K. Chayamarit et al. 2595* (BKF); • Khlong Phanom National Park, fr., 21 Mar 2001 (as *Garcinia* sp.), *D. J. Middleton et al. 545* (A [A00466338], BKF); Khao Sok National Park, fl., 10 May 2002 (as *Garcinia* sp.), *R. Pooma et al. 3700* (BKF); • Khlong Yan Wildlife Sanctuary, Vibhavadi District, ♀ fl., 31 Aug. 2002, *D. J. Middleton et al. 1479* (BKF, CMUB, E [E00351023], K, L [L3881861]); • Ko Mae Ko, Mu Ko Ang Thong National Park, ♀ fl., fr., 29 Mar 2025, *C. Ngernsaengsaruay* personal observation with photos]; • Phangnga [Takua Pa District, fl., 16 Feb 1929 (as *Garcinia* sp.), *A. F. G. Kerr 17101* (BK, BM, K); • Foothill of Khao Phra Mi, young fr., 7 Jan 1966 (as *Garcinia* sp.), *B. Hansen & T. Smitinand 11811* (C, K, SING); ibid., fl., 11 Jul 1972, *K. Larsen et al. 30881* (AAU, BKF, K, L [L.2416503], P [P05061540], SING); • N. of Thung Maphrao, fl., 19 Jul 1972, *K. Larsen et al. 31127* (AAU, BKF, C, E [E00839780], K, L [L2416517], P [P05061536]); • Khlong Nang Yon, fr., 28 Apr 1973 (as *Garcinia* sp.), *R. Geesink & T. Santisuk 5000* (AAU, BKF, C, K, P [P05062053]); • ibid., ♀ fl., 30 Apr 1973 (as *Garcinia* sp.), *R. Geesink & T. Santisuk 5064* (AAU, BKF 57599, C, K, P [P05062014]); • ibid., fr., 25 Nov 1974 (as *Garcinia* sp.), *R. Geesink et al. 7600* (AAU, BKF, C, K, P [P05062001]); • Ko Surin Nuea, fl., 13 Apr 1976, *C. Chermsirivathana & T. Smitinand 2053* (BK, BKF); • ibid., fr., 15 Apr 1976, *C. Chermsirivathana & T. Smitinand 2092* (BK, BKF); • Khura Buri District, fr., 10 Dec 1976, *T. Santisuk 809* (PSU); • Suan Wang Temple, Khura Buri District, ♂ fl., 28 Nov 2019, *C. Ngernsaengsaruay G63-28112019* (BKF); • Ko Kho Khao, ♂ fl. (spirit collection), 18 Jun 2009, *C. Ngernsaengsaruay G62-18062009* (BKF); • Ko 4, Similan National Park, ♂ fl., 2 Dec 1992, *C. Niyomdham & P. Puudjaa 3407* (AAU, BKF); • Ko Bon, fr., 8 Apr 1999, *T. Wongprasert s.n.* (BKF127989); • Khao Lak-Lam Ru National Park, Takua Pa District, fr., 12 Apr 2003 (as *Garcinia* sp.), *D. J. Middleton et al. 2159* (A [A00466330], BKF); • Chang Fa Waterfall, Khao Lak-Lam Ru National Park, Takua Pa District, fl., 12 Jun 2004 (as *Garcinia* sp.), *S. Gardner & P. Sidisunthorn ST0730* (K); • Ton Pling Waterfall, Khao Lak-Lam Ru National Park, Thai Mueang District, fr., 20 Oct 2004 (as *Garcinia* sp.), *S. Gardner & P. Tippayasri ST0749* (K), *ST0749a* (K); • Si Phangnga National Park, Khura Buri District, fl., 11 Dec 2003 (as *Garcinia* sp.), *A. S. Barfod et al. 558* (AAU); • ibid., fl., 24 Jun 2004 (as *Garcinia* sp.), *S. Gardner & P. Sidisunthorn ST0849* (K); • Ko Phra Thong, Khura Buri District, fl., 26 Apr 2005 (as *G. acuminata*), C. Phengklai et al. 15004 (BKF); • Bangwan Stream, Khura Buri District, ♀ fl. & fr., 26 Nov 2006 (as *G. eugeniifolia*), *T. Muadsub 164* (BKF, PSU)]; • Phuket [Hill near Thalang District, fr., 8 May 1968 (as *Garcinia* sp.), *C. F. van Beusekom & C. Phengkhlai 657* (AAU, C, K, P [P05062038]); • Khao Phra Thaeo, ♂ fl., 12 Jul 1979 (as *Garcinia* sp.), *C. Niyomdham et al. 291* (AAU, BKF, C, K, P [P05061703]); • Ton Sai Waterfall, fr., 17 Mar 1981 (as *Garcinia* sp.), *G. Congdon 1310* (PSU); • Locality unspecified, fr., 8 Aug 2017, *U. Veesommai s.n.* (BK)]; • Krabi [Ao Luk District, ♂ fl., 14 Mar 1930, *A. F. G. Kerr 18558* (BK, BM, C, E [E00839781], K, L [L2416508], P [P05061544]); • Than Bok Khorani National Park, fl., 10 May 1973 (as *Garcinia* sp.), *R. Geesink & T. Santisuk 5320* (BKF); • Ko Phi Phi, fl., 9 Apr 1930, *A. F. G. Kerr 18894* (AAU, BK, BM, E [E00839783], K); • ibid., fr., 9 Apr 1930, *A. F. G. Kerr 18900* (BM, E [E00839785], K); • Khao Pra Bang Khram Wildlife Sanctuary, Khlong Thom District, ♂ fl., 5 Apr 1988 (as *G. rostrata*), *C. Niyomdham & W. Ueachirakan 1774* (AAU, BKF 089423, C, K, L [L2417529], P [P04701340]); • ibid., fr., 5 Oct 2005, *J. F. Maxwell 05-542* (BKF, CMUB, L [L3878267, L3878268]); • ibid., ♂ fl., 23 Mar 2006, *J. F. Maxwell 06-177* (CMUB, L [L3878638], QBG); • ibid., sterile, 15 Feb 2022, *C. Ngernsaengsaruay & W. Boonthasak G64-15022022* (BKF); • Khao No Chuchi, fr., 28 Feb 1994 (as *Garcinia* sp.), *A. S. Barfod et al. 45276* (BKF, K); • Khao Phanom Bencha National Park, 14 Feb 2022, *C. Ngernsaengsaruay* personal observation with photos]; • Nakhon Si Thammarat [Ao Wang Khram, Thung Song District, young fr., 25 Jul 1929, *Rabil 200* (AAU, BK, BM, E [E00839789], K); • Chawang District, fr., 20 Feb 1957 (as *Garcinia* sp.), *Snan 922* (BKF); • Karom Waterfall, Khao Luang National Park, Lan Saka District, fr., 23 Aug 1980 (as *G. eugeniifolia*), *P. Sirirugsa 331* (PSU); • ibid., ♂ fl., 13 Apr 1985, *J. F. Maxwell 85-385* (AAU, BKF, E [E00839787], L [L2416499], PSU); • ibid., ♂ fl., 16 Nov 1985, *J. F. Maxwell 85-1026* (BKF, E [E00839782], L [L2416516], PSU)]; • Phatthalung [Khao Ok Thalu, ♂ fl., 21 Apr 1928, *A. F. G. Kerr 15337* (BK, BM, K); • Si Nakharin roadside Trang to Phatthalung, fr., 25 Dec 2006 (as *Garcinia* sp.), *R. Pooma et al. 6619* (BKF)]; • Trang [Khao Soi Dao, ♂ fl., 28 Apr 1930, *A. F. G. Kerr 19180* (BK, BM, C, K, L [L2416509], P [P05061546]); • Kachong, ♀ fl., April 1949 (as *G. rostrata*), *S. Boonkerd 42* (BKF); • ibid., ♂ fl., 5 April 1949 (as *G. rostrata*), *S. Boonkerd 52* (BKF); • ibid., 17 April 1949 (as *G. rostrata*), *S. Boonkerd 71* (BKF); • Khao Chong, fl., 25 Jan 1958 (as G. cf. rostrata), *T. Smitinand 4099* (BKF); • ibid., 13 Oct 1965 (as *Garcinia* sp.), *C. Boonnab 65* (BKF); • ibid., fl. 12 Apr 1966 (as *Garcinia* sp.), *C. Boonnab 473* (BKF); • ibid., fr., 1 Feb 1969, *S. Phusomsaeng & S. Pinnin 46* (BKF, C, K); • ibid., ♂ fl., 20 Apr 1969, *S. Phusomsaeng 217* (BKF, C, K, L [L2416511]); • ibid., fl., 18 Jun 1969 (as *Garcinia* sp.), *B. Sangkhachand 1883* (BK); • ibid., fl., 22 Jun 1969 (as *Garcinia* sp.), *B. Sangkhachand 1901* (BK); • ibid., fl., 1 Jul 1969 (as *Garcinia* sp.), *B. Sangkhachand 1945* (BK); • ibid., ♀ fl., 6 Apr 1971 (as *Garcinia* sp., *G. eugeniifolia*), *S. Phusomsaeng 419* (BKF, C, K, P [P05062018]); • ibid., young fr., 13 Aug 1975, *J. F. Maxwell 75-817* (AAU, BK, L [L2416501]); • ibid., ♂ fl., 18 Jun 1987, *J. F. Maxwell 87-580* (AAU, BKF, L [L2416518], PSU, P [P05061537]); • ibid., fr., 24 Mar 1993 (as *Garcinia* sp.), *P. Chantaranothai et al. 1342* (K); • Khao Chong 16-hectare plot, fl., Feb 2001 (as *Garcinia* sp.), *A. Sinbumroong & S. Davies AS211* (BKF); • ibid. fr., Feb 2001 (as *Garcinia* sp.), *A. Sinbumroong & S. Davies AS229* (BKF); • Khao Chong, Chong Subdistrict, Na Yong District, fr., 16 Feb 2022, *C. Ngernsaengsaruay & W. Boonthasak G65-16022022* (BKF); • ibid., fr. (spirit collection), 17 Mar 2004, *C. Ngernsaengsaruay G59-17032004* (BKF); • Thung Khai Botanical Garden, Thung Khai Subdistrict, Yan Ta Khao District, ♂ fl. (spirit collection), 18 Mar 2004, *C. Ngernsaengsaruay G60-18032004* (BKF); • Locality unspecified, fr., 4 Dec 1971 (as *G. rostrata*), *S. Pinnin et al. 318* (BKF, C); • Locality unspecified, young fr., 13 Jun 1973 (as *Garcinia* sp.), *S. Phusomsaeng et al. 1609* (BKF, C, K, P [P05062013]); • Locality unspecified, fr., 1 Dec 1974 (as *Garcinia* sp., *G. acuminata*), *B. Nimanong & S. Phusomsaeng 1608* (BKF, C, K, P [P05062043]); • Peninsular Botanic Gardens, Thung Khai, ♀ fl. & fr., 6 Apr 1988 (as *G. rostrata*), *C. Niyomdham & W. Ueachirakan 1779* (AAU, BKF, C, K, L [L2417530], P [P04701339]); • ibid., fl., 21 Feb 1995, *N. Chintana 13* (BKF); • ibid., young fr., 2 Sep 1997, *V. Chamchumroon et al. 1/3* (BKF); • ibid., fr., 23 Jan 1997, *V. Chamchumroon 3/4* (BKF); • ibid., fl. & fr., 16 Jan 2001, *S. Praknuk et al. 6* (BKF); • ibid., fr., 24 Apr 2001, *S. Praknuk et al. 3* (BKF); • ibid., fr., 24 Apr 2001, *S. Praknuk et al. s.n.* (BKF131169); • ibid., fl., 22 Feb 2004 (as *Garcinia* sp.), *S. Gardner & P. Sidisunthorn ST0060* (K); • ibid., fr., 23 Feb 2004 (as *Garcinia* sp.), S. *Gardner & P. Sidisunthorn ST0069* (K); • ibid., fr., 17 Nov 2012 (as *Garcinia* sp.), *V. Chamchumroon et al. 5670* (BKF); • Sai Rung Waterfall, fr., May 2001 (as *Garcinia* sp.), *A. Sinbumroong & S. Davies AS6* (A [A00466128], BKF); • Khao Banthat, Yan Ta Khao District, fl., 7 Apr 2003 (as *Garcinia* sp.), *D. J. Middleton et al. 1986* (A [A00466336], BKF)]; • Satun [Adang, fr., 12 Jan 1928, *A. F. G. Kerr 14017* (C, K, L [L2416469], P [P05061547]); • Ao Son, Tarutao National Park, La Ngu District, fl., 10 Feb 2005 (as *Garcinia* sp.), *P. Sidisunthorn & P. Tippayasri ST1484* (K); • Ko Tarutao, fl., 8 Apr 2008 (as *Garcinia* sp.), *C. Phengkhlai 15728* (BKF); • ibid., fl., 7 Apr 2008, *B. Sonsupab 3899* (BK); • ibid., fr., 9 Apr 2008, *B. Sonsupab 3903* (BK)]; • Songkhla [Ton Nga Chang Wildlife Sanctuary, Hat Yai District, ♂ fl., 2 Oct 1984 (as *Garcinia* sp.), *J. F. Maxwell 84-273* (P [P04899144], PSU); • ibid., ♂ fl., 22 Jan 1985, *J. F. Maxwell 85-88* (PSU); • Suan Toon Waterfall, ♂ fl., 12 Feb 1985, *J. F. Maxwell 85-180* (BKF, P [P04788046], PSU); • Khao Khlong Rang, Na Mom District, ♀ fl., 15 Feb 1986, *J. F. Maxwell 86-66* (AAU, BKF, E [E00839784], L [L2416497], PSU)]; • Pattani [Kola Po, ♀ fl., 5 Apr 1928, *A. F. G. Kerr 15072* (AAU, BK, BM, C, E [E00160910], K)]; • Yala [Locality unspecified, ♂ fl., 29 Jan 1931, *Put 3656* (AAU, BK, BM, K); • Khao Joh Tong, Wang Sai, Betong District, fr., 22 May 2000 (as *G. eugeniifolia*), *C. Niyomdham 6169* (AAU, BKF)]; • Narathiwat [Bacho District, fr., 22 Nov 1961, *B. Sangkhachand 258* (BKF, K, L [L2416512]); • Ra-ngae District, 16 Dec 1961 (as *Garcinia* sp.), *B. Sangkhachand 933* (C, K, P); • Khlong Ai Kading, Hala Bala Wildlife Sanctuary, fr., 21 Feb 2003, *C. Niyomdham & P. Puudjaa 7076* (BKF); • Hala Bala Wildlife Sanctuary, fr., 22 Feb 2005, *C. Niyomdham & P. Puudjaa 7271* (BKF); • Khlong Saphan Song, Hala Bala Wildlife Sanctuary, Waeng District, fl., 28 Apr 2011 (as *Garcinia* sp.), *P. Puudjaa et al. 1689* (BKF)]; • Region and Province unspecified [Locality unspecified, s.d., *A. F. G. Kerr 61* (C)].

**Figure 1.**
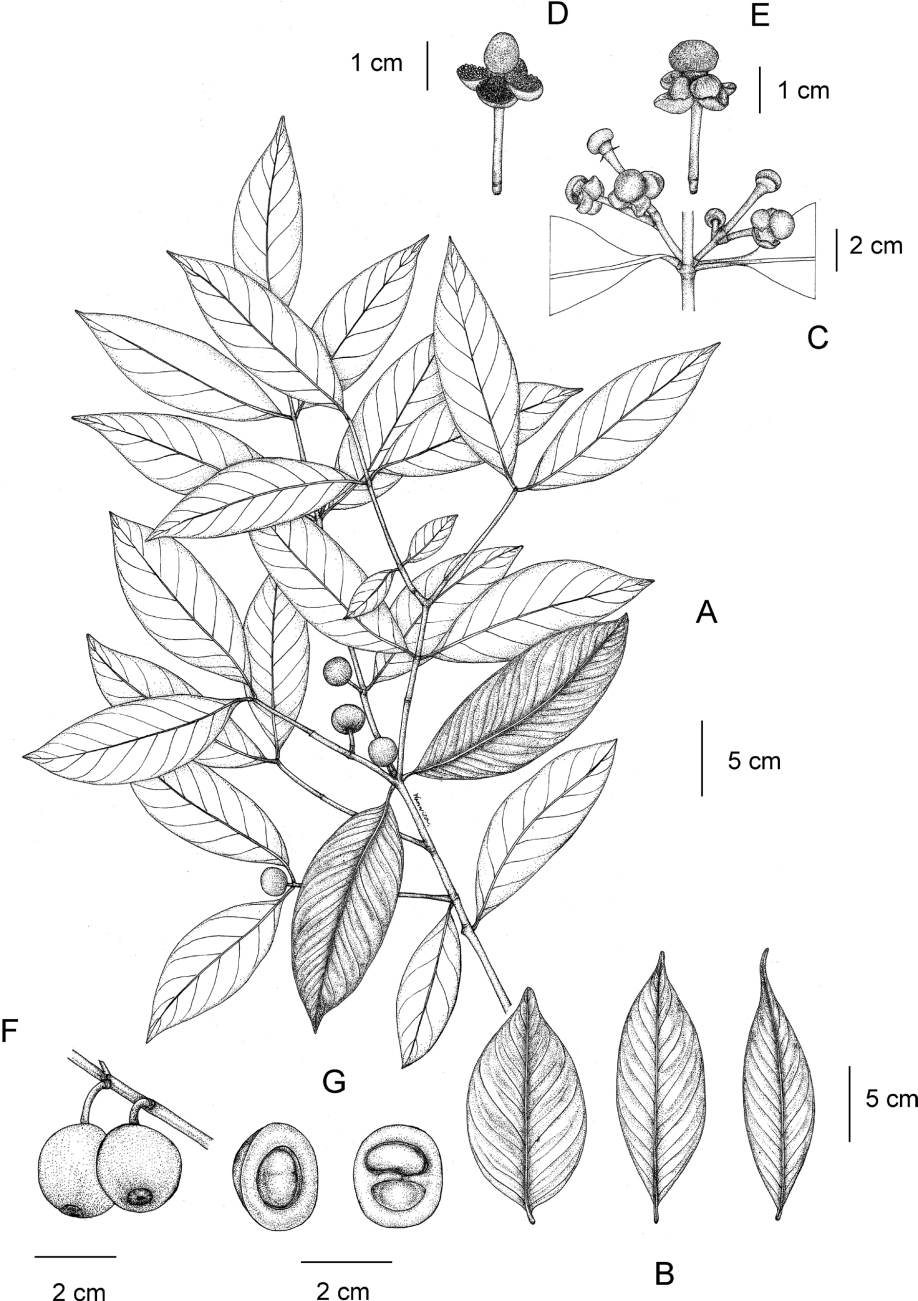
*
Garcinia merguensis*. A. Branchlets, leaves, and fruits; B. Variation in leaf shape and apex; C. Branchlets, inflorescences with female flowers; D. Male flower; E. Female flower; F. Branchlet and fruits; G. Fruit and seeds. Photo: Drawn by Wanwisa Bhuchaisri.

**Figure 2.**
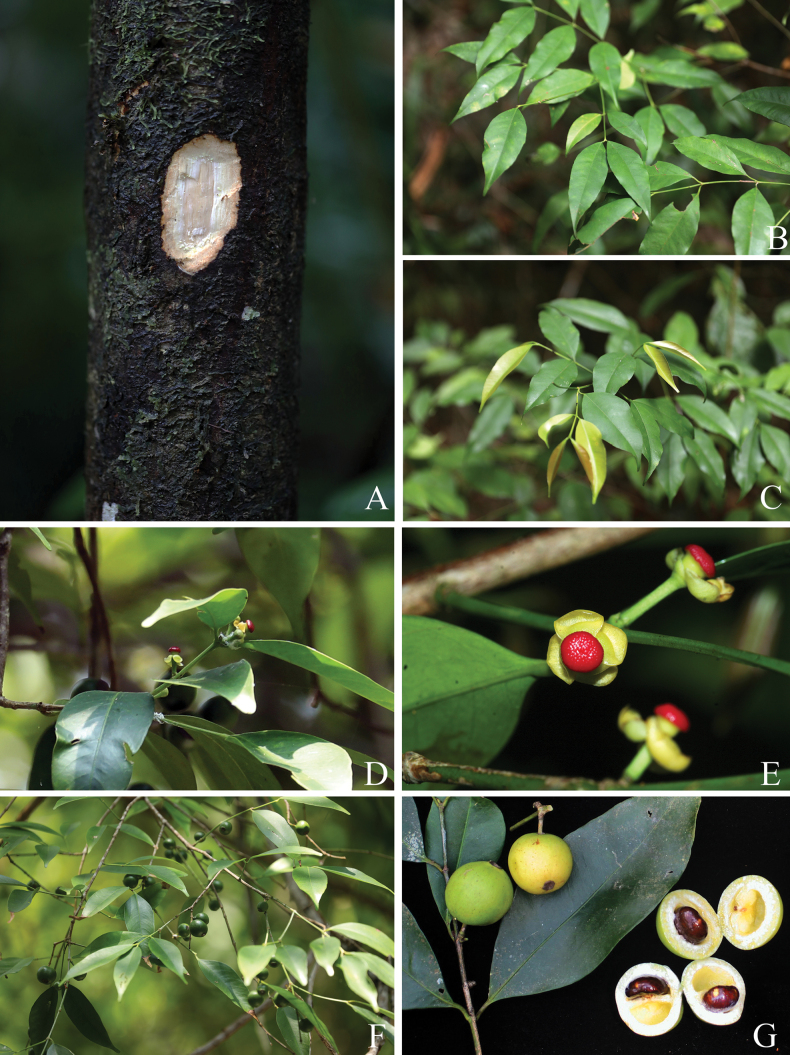
*
Garcinia merguensis*. A. Slashed bark with white exudate; B, C. Branchlets, mature and young leaves; D, E. Branchlets, leaves, and female flowers; F. Branchlets, leaves, and fruits; G. Mature and ripe fruits and seeds. Photos: Chatchai Ngernsaengsaruay.

**Figure 3.**
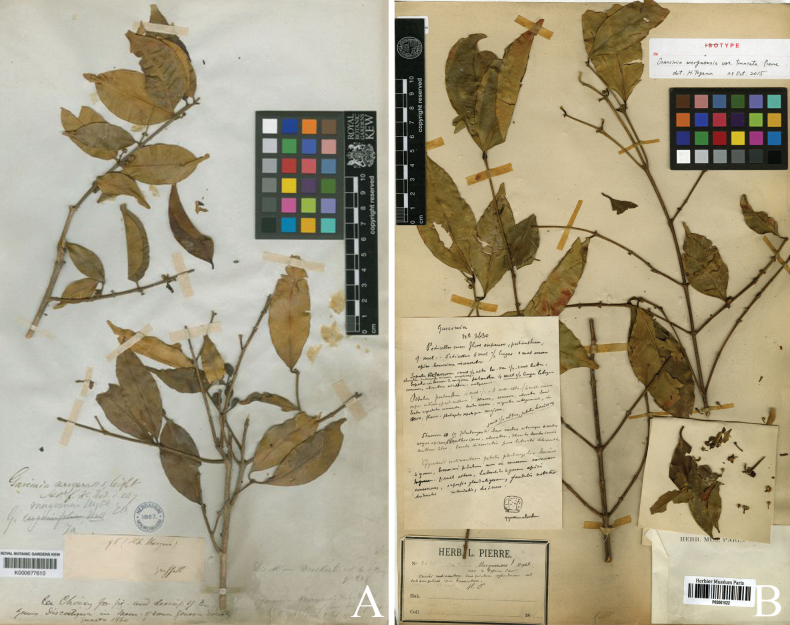
*
Garcinia merguensis*. A. Lectotype of *Garcinia merguensis*, *W. Griffith 96* (K [K000677610]) from Mergui, Myanmar, designated by Maheshwari (1964); B. Lectotype of Garcinia merguensis var. truncata, a synonym of *Garcinia merguensis*, *J. B. L. Pierre 3630* (P [P05061522]) from ad Cay Cong in prov. Tayninh gallicae austro Cochinchinae, Vietnam, designated here. Photos: The Board of Trustees of the RBG, Kew, http://specimens.kew.org/herbarium/K000677610 (A), Muséum National d’Histoire Naturelle, Paris, France (MNHN), http://coldb.mnhn.fr/catalognumber/mnhn/p/p05061522 (B).

**Figure 4.**
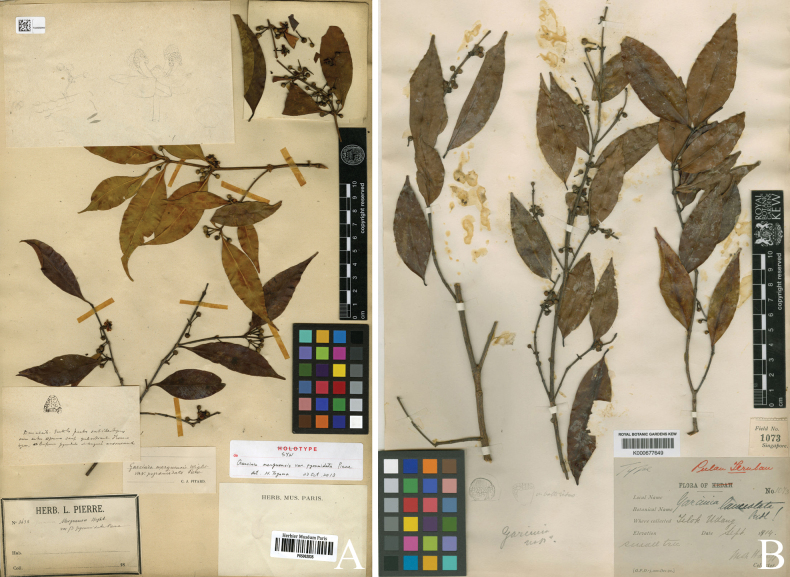
*
Garcinia merguensis*. A. Lectotype of Garcinia merguensis var. pyramidata, a synonym of *Garcinia merguensis*, *J. B. L. Pierre 3638* (P [P05062835]) from ad montem Cam Chay in prov. Kampot, Cambodia, designated here; B. Lectotype of *Garcinia lanceolata*, a synonym of *Garcinia merguensis*, *M. Haniff 1073* (K [K000677649]) from Langkawi, Pulau Terutau, Telok Udang, Malaysia, designated here. Photos: Muséum National d’Histoire Naturelle, Paris, France (MNHN), http://coldb.mnhn.fr/catalognumber/mnhn/p/p05062835 (A), The Board of Trustees of the RBG, Kew, http://specimens.kew.org/herbarium/K000677649 (B).

**Figure 5.**
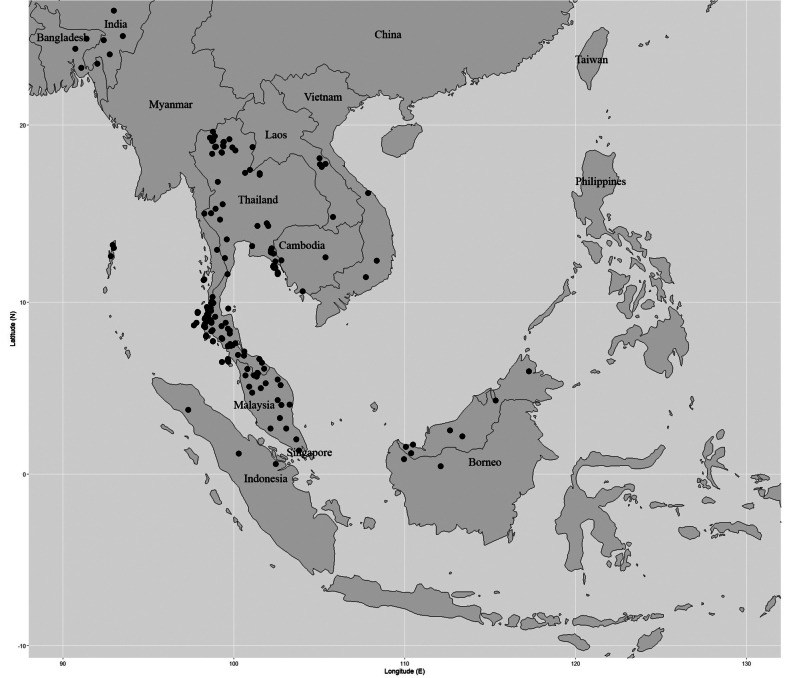
Distribution of *Garcinia merguensis*. It is known from India (Assam, Andaman, and Nicobar Islands), Bangladesh, Myanmar (Tenasserim, Mergui Archipelago), Sumatra, and Borneo. In Thailand, this species is found in all floristic regions. Map: Pichet Chonton and Chatchai Ngernsaengsaruay.

#### ﻿2. Garcinia minutiflora

Taxon classificationPlantaeMalpighialesClusiaceae

Ridl., J. Straits Branch Roy. Asiat. Soc. 82: 169. 1920

11FCE676-992E-5085-A6C2-93BC9F9885D0

Fig. 6.

Garcinia minutiflora Ridl., J. Straits Branch Roy. Asiat. Soc. 82: 169. 1920; Fl. Malay Penins. 1: 176. 1922; Craib, Fl. Siam. 1(1): 117. 1925; S. C. Chin, Limestone Fl. Malaya, M.Sc. Thesis, Univ. Malaya: 287. 1973; Kochummen & Whitmore, Gard. Bull. Singapore 26(2): 272. 1973; Whitmore in Whitmore, Tree Fl. Malaya 2: 216. 1973; I. M. Turner, Gard. Bull. Singapore 47(1): 262. 1995; Ngerns. et al., Thai Forest Bull., Bot. 52(2): 73. figs. 1, 2, and 3. 2024.

##### Type.

***Lectotype*** [designated by Ngernsaengsaruay et al. (2024b)], • Peninsular Malaysia, Langkawi Island, Goa Cherita (original publication “Goa Chinta”), ♂ fl., Mar 1892, *C. Curtis 2802*, K digital image! [K000677659]; ***isolectotypes***: SING! [SING0063116, SING0063117].

##### Description.

***Habit*** trees, 3–8(–15) m tall, 20–40(–60) cm GBH; exudate yellow, sticky; branchlets 4-angular, glabrous. ***Bark*** dark greyish brown to dark brown, smooth, rough, or scaly. ***Leaves*** obovate or elliptic, 3.8–6 × 1.7–3.7 cm, apex obtuse or retuse, base cuneate or obtuse, margin entire and finely revolute, coriaceous, dark green above, paler below, glabrous on both surfaces, midrib slightly raised above, raised below, secondary veins 5–9 on each side, curving towards the margin and connected in distinct loops and united into an intramarginal vein, conspicuous on both surfaces, with intersecondary veins, veinlets reticulate, conspicuous on both surfaces, with many scattered black gland dots below, interrupted long wavy lines present, of differing lengths, nearly parallel to the midrib, running across the secondary veins to the apex or the margin, visible below; petiole 0.3–1 cm long, 1–2 mm diam., grooved above, glabrous, with a small basal appendage clasping the branchlet; fresh leaves brittle when crushed; young leaves pale green and petiole red or reddish green; dry leaves brown or brownish green. ***Inflorescences*** axillary or on branchlets at leafless nodes (in axils of fallen leaves), in short thyrses of 5–12 flowers, 2–3-flowered cymes, or solitary. ***Flowers*** unisexual; bracteoles 4, decussate; sepals and petals decussate, glabrous. ***Flower buds*** pale green, subglobose or globose, c. 2 mm diam. ***Male flowers*** in a short thyrse, 0.8–2 cm long, 5–12 flowers, small, 1.8–2 mm diam.; bracteoles semi-orbicular, 0.7–1.1 × 0.9–1 mm, apex obtuse; pedicel pale green, 1.4–1.5 mm long, 1.7–1.8 mm diam., glabrous; sepals 4, pale green, concave, the outer pair slightly larger than the inner pair, the outer pair suborbicular or broadly ovate, 1.6–2 × 1.6–2 mm, apex obtuse, the inner pair ovate or suborbicular, 1–1.5 × 1–1.5 mm, apex obtuse; petals 4, creamish white or pale yellow, obovate, 1–1.5 × 0.8–1.1 mm, subequal, apex obtuse or rounded, slightly smaller than the sepals; stamens numerous, filaments completely united into 4 bundles, each bundle 0.4–0.5 × 0.3–0.5 mm; anthers globose or subglobose, 0.1–0.2 × c. 0.1 mm; pistillode mushroom-shaped, 0.5–0.7 mm long; sterile stigma sessile, slightly convex, c. 0.3 mm diam., weakly 4–5-lobed, papillate. ***Female flowers*** not seen. ***Fruits*** berries, subglobose, globose, or broadly ellipsoid, 1.4–1.7 × 1.3–1.5 cm, green, smooth with fine longitudinal striate, glabrous, pericarp coriaceous, c. 0.6 mm thick, cut fruits with a sticky yellow exudate, with small persistent sepals; persistent stigma blackish brown or dark brown, convex, 2–3 mm diam., weakly 4–5-lobed, papillate; fruiting stalk 2–3 mm long, 1.5–3 mm diam. ***Seeds*** 1–2, black when dry, depressed subglobose, c. 1.1 × 1.2 cm, c. 7.8 mm thick, with a thin fleshy pulp.

##### Distribution.

Peninsular Thailand, Peninsular Malaysia [Perlis, Kedah (Langkawi Island), Penang, Kelantan].

##### Distribution in Thailand.

**Peninsular**: Phangnga, Krabi.

##### Habitat and ecology.

This species is found in littoral dry evergreen forest on limestone hills and dry evergreen forest on limestone hills, at altitudes reaching up to 250 m a.m.s.l.

##### Phenology.

Flowering and fruiting more than once a year, from August to March.

##### Conservation status.

Rare in Peninsular Malaysia (Ridley 1922). In Thailand, the species is known only from three locations in Phangnga and Krabi Provinces but is expected in other limestone hills. Globally, this species is known from Peninsular Malaysia and Peninsular Thailand and has a small EOO of 6,774.65 km^2^ and a relatively small AOO of 28 km^2^ that lies within protected and non-protected areas. It is inferred to be experiencing a continuing decline in habitat area, extent, and quality. We therefore consider the conservation assessment as Vulnerable [VU B1a,b(iii),B2a,b(iii)].

##### Etymology.

The specific epithet of *Garcinia minutiflora* comes from the Latin compound words *minutus*, meaning very small or minute, and -*flora*, *flos*, meaning flower, referring to the very small flowers (Stearn 1992; Radcliffe-Smith 1998; Gledhill 2002).

##### Vernacular name.

**Nuan dok lek khao hin pun** (**นวลดอกเล็กเขาหินปูน**) (Ngernsaengsaruay et al. 2024b).

##### Uses.

Not known.

##### Notes.

The morphological characters and data reported here for this species were mostly taken from Ngernsaengsaruay et al. (2024b).

According to Chin (1973), the female flowers of *Garcinia minutiflora* are solitary or in 2-flowered cymes, but we have not seen them.

Craib (1925) reported *Garcinia minutiflora* from the former Phuket (then consisting of present-day Ranong, Phangnga, Phuket, Krabi, Trang, and Satun Provinces). He cited only the type *C. Curtis 2802* but did not mention any specimens from Thailand. Langkawi Island (Ko Langkawi) is an administrative district of Kedah located about 30 km off the coast of northwestern Peninsular Malaysia and a few kilometres south of Ko Tarutao (Tarutao Island), Satun Province, adjacent to the Thai border (Ngernsaengsaruay et al. 2024b).

According to previous studies, the male flowers of *Garcinia minutiflora* have numerous stamens in a central globose mass, without a pistillode (Ridley 1920, 1922; Kochummen and Whitmore 1973; Whitmore 1973). However, from our examinations, we found the stamens are numerous, and the filaments are completely united in 4 bundles surrounding a pistillode. In the early stage of open flowers, the stamen bundles are pressed against the pistillode (not spreading) and then become spreading in the fully open flowers.

According to Ngernsaengsaruay et al. (2024b), a preliminary conservation assessment of *Garcinia minutiflora* was considered as VU [B1B2a,b(ii,iv)]. Because it is a limestone species with a narrow geographic range and because of its small number of locations and small AOO, this species is evaluated as VU [B1a,b(iii),B2a,b(iii)] in contrast with Chua (2023) as LC.

##### Additional specimens examined.

**Thailand. Peninsular**: • Phangnga [Khao Phing Kan (Ko Khao Phing Kan), Takua Thung District, fr., 8 Sep 1982 (as *Garcinia* sp.), *T. Shimizu et al. T-29205* (BKF)]; • Krabi [Wat Tham Suea, Mueang Krabi District, fr., 18 Apr 2007 (as *Garcinia* sp.), *S. Gardner ST2893* (K); • ibid., sterile, 7 Mar 2022, *C. Ngernsaengsaruay & W Boonthasak G29-07032022* (BKF); • ibid., fl., 7 Mar 2022, *C. Ngernsaengsaruay & W. Boonthasak* personal observation, with photos; • Ko Hong, trail up to view point, Than Bok Khorani National Park, Mueang Krabi District (originally “Koh Hong, Ao Luek District” on the label), fr., 15 Mar 2021 [as *Garcinia* sp.], *N. Tetsana et al. 2087* (BKF); • ibid., fl. buds, 15 Mar 2021 (as *Garcinia* sp.), *N. Tetsana et al. 2088* (BKF)].

**Figure 6.**
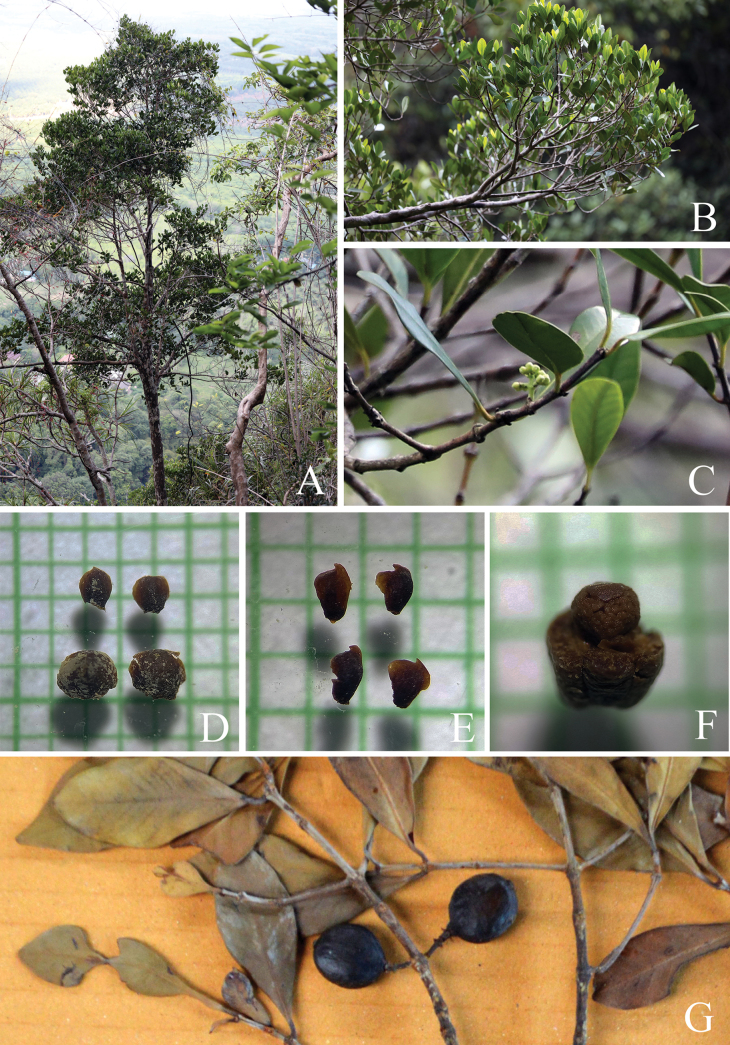
*
Garcinia minutiflora*. A. Habitat and habit; B. Branch, branchlets, and leaves; C. Branchlets, leaves, and inflorescences with male flower buds; D. Sepals; E. Petals; F. Male flowers (sepals and petals removed); in the early stage of open flower, the stamen bundles are pressed against the pistillode (not spreading); G. Branchlets, leaves, and fruits. Photos: Chatchai Ngernsaengsaruay (A–C); Weereesa Boonthasak (D–G).

#### ﻿3. Garcinia rostrata

Taxon classificationPlantaeMalpighialesClusiaceae

(Hassk.) Miq., Ann. Mus. Bot. Lugduno-Batavi 1(7): 209. 1864

3D6F2B05-F97D-5835-AA36-E6A967A12C74

**Figure 7.**
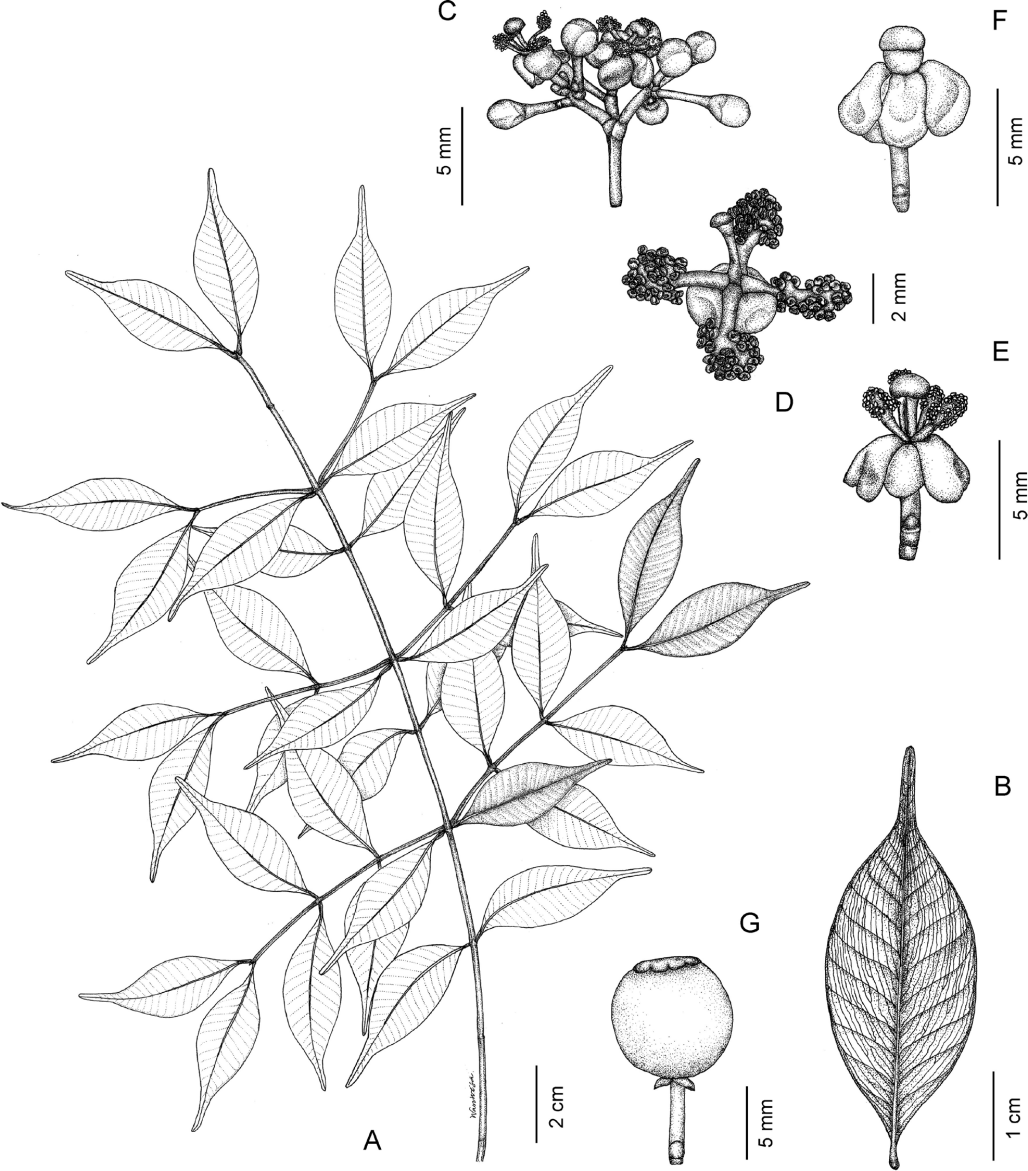
*
Garcinia rostrata*. A. Branchlets and leaves; B. Leaf; C. Inflorescence with male flowers; D. Male flower (top view); E. Male flower (side view); F. Female flower; G. Fruit. Photo: Drawn by Wanwisa Bhuchaisri.

**Figure 8.**
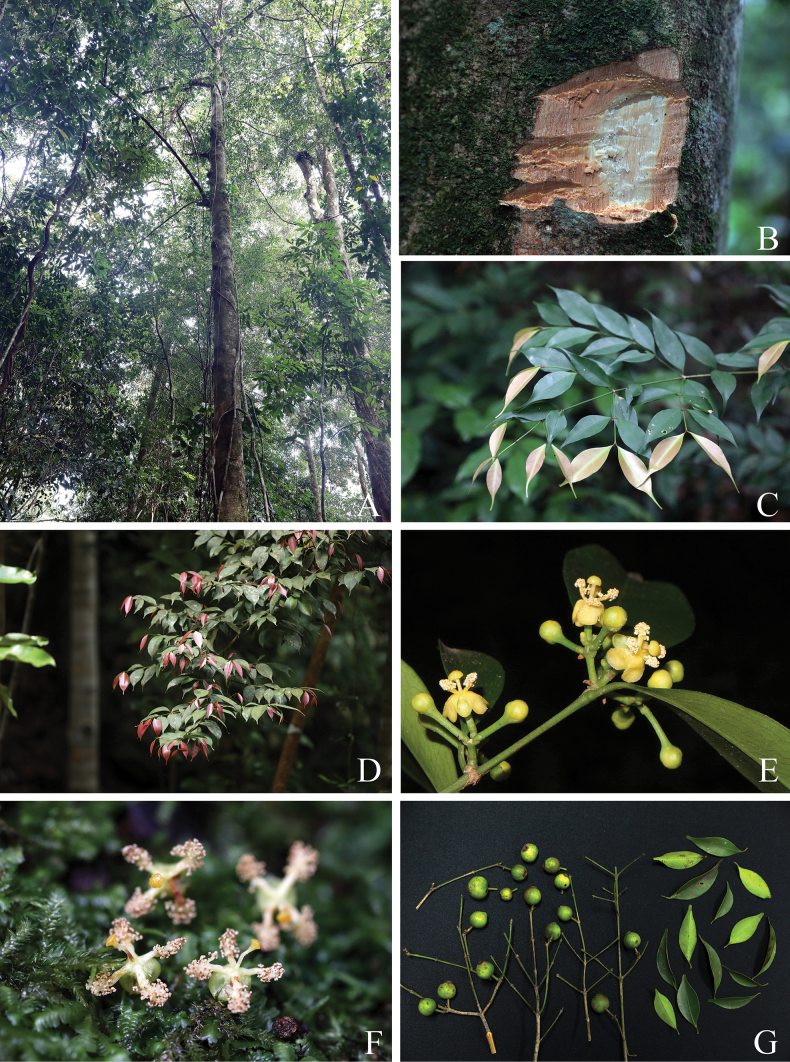
*
Garcinia rostrata*. A. Habit and habitat; B. Slashed bark with creamish white exudate; C, D. Branchlets, mature and young leaves; E. Branchlets and inflorescences with male flowers; F. Male flowers; G. Branchlets, leaves, and fruits. Photos: Chatchai Ngernsaengsaruay (A–D, F, G); Pichet Chanton (E).

**Figure 9.**
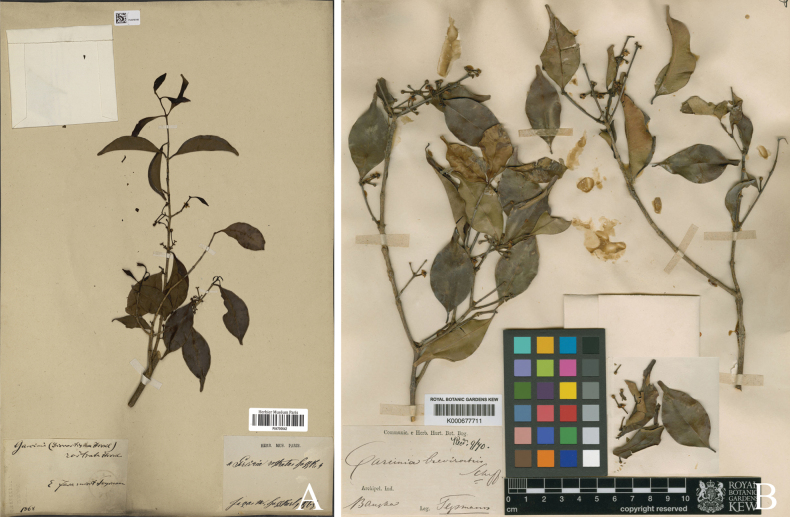
*
Garcinia rostrata*. A. Lectotype of *Garcinia rostrata*, *J. E. Teijsmann 1868* (P [P04700642]) from Java, Indonesia, designated here; B. Isolectotype of *Garcinia brevirostris*, a new synonym of *Garcinia rostrata*, *J. E. Teijsmann s.n.* (K [K000677711]) from Djeboes, Bangka, Sumatra, Indonesia, designated here. Photos: Muséum National d’Histoire Naturelle, Paris, France (MNHN), http://coldb.mnhn.fr/catalognumber/mnhn/p/p04700642 (A), The Board of Trustees of the RBG, Kew, http://specimens.kew.org/herbarium/K000677711 (B).

**Figure 10.**
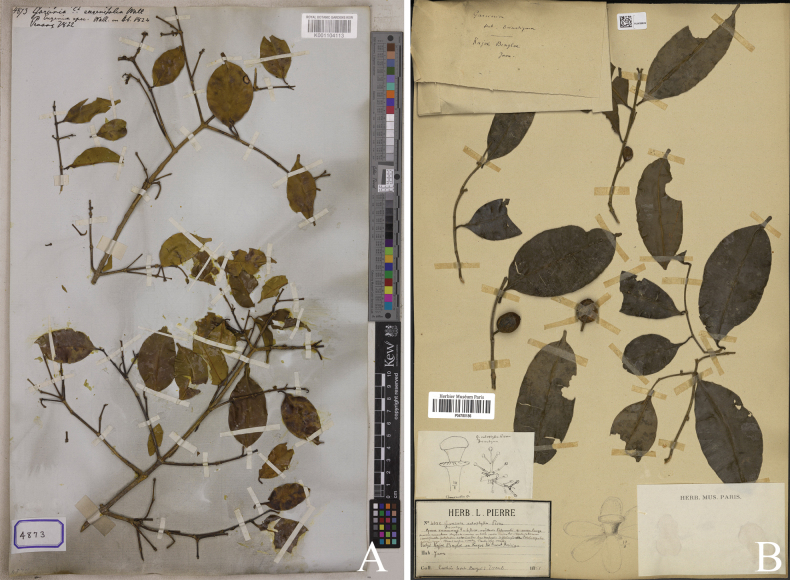
*
Garcinia rostrata*. A. Isolectotype of *Garcinia eugeniifolia*, a synonym of *Garcinia rostrata*, *Wallich Cat. 4873* (K [K001104113]) from Penang, Peninsular Malaysia, designated by Maheshwari (1964); B. Lectotype of *Garcinia calophylla*, a new synonym of *Garcinia rostrata*, *Treub s.n.* (*Herb. L. Pierre 4632*) (P [P04700186]) cultivated in Hort. Bot. Bogor., Java, Indonesia, designated here. Photos: The Board of Trustees of the RBG, Kew, http://specimens.kew.org/herbarium/K001104113 (A), Muséum National d’Histoire Naturelle, Paris, France (MNHN), http://coldb.mnhn.fr/catalognumber/mnhn/p/p04700186 (B).

**Figure 11.**
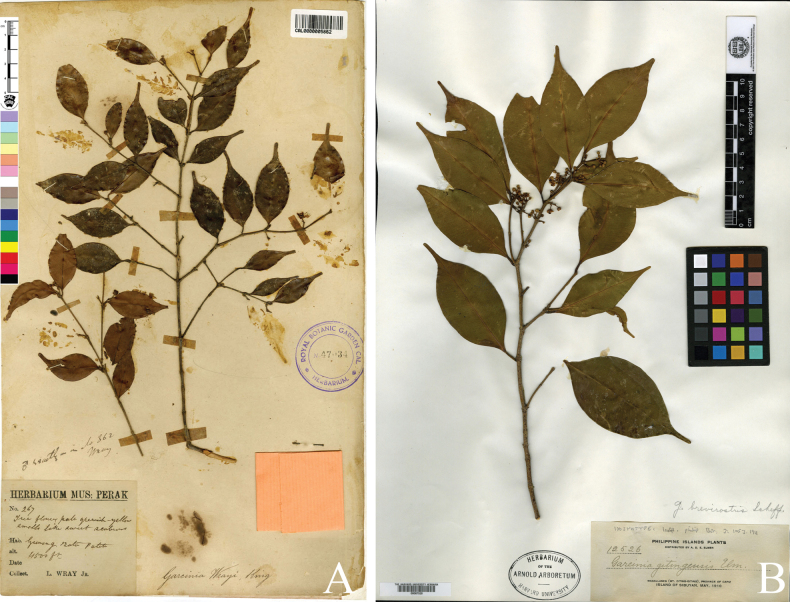
*
Garcinia rostrata*. A. Lectotype of *Garcinia wrayi*, a synonym of *Garcinia rostrata*, *L. Wray 267* (CAL [CAL0000005862]) from Gunong Batu Pateh, Perak, Peninsular Malaysia, designated here; B. Lectotype of *Garcinia gitingensis*, a synonym of *Garcinia rostrata*, *A. D. E. Elmer 12526* (A [A00067528]) from Magallanes (Mt. Giting-giting), Province of Capiz, Island of Sibuyan, Philippines, designated here. Photos: Botanical Survey of India, http://ivh.bsi.gov.in/phanerogams-Details/en?link=CAL0000005862&column=szBarcode (A), Harvard University Herbaria, Cambridge, Massachusetts, U.S.A. https://kiki.huh.harvard.edu/databases/specimen_search.php?barcode=A000675 (B).

Figs 7 , 8 , 9 , 10 , 11

Garcinia rostrata (Hassk.) Miq., Ann. Mus. Bot. Lugduno-Batavi 1(7): 209. 1864; Kurz, Forest Fl. Burma 1: 89. 1877; Pierre, Fl. Forest. Cochinch. 1(5): 5. t. 91B. 1883; King, J. Asiat. Soc. Bengal, Pt. 2, Nat. Hist. 59(2): 151. 1890; Engl. in Engl. & Prantl, Die Naturlichen Pflanzenfamilien 3(6): 236. 1893; Vesque, Epharmosis 2: 15. t. 93. 1889 et in A. DC. & C. DC., Monogr. Phan. 8: 340. 1893; Koord. & Valeton, Bijdr. Boomsoort. Java 9: 361. 1903; Ridl., Fl. Malay Penins. 1: 169. 1922; Backer & Bakh. f., Fl. Java (Spermatoph.) 1: 388. 1963; Kochummen & Whitmore, Gard. Bull. Singapore 26(2): 277. 1973; Whitmore in Whitmore, Tree Fl. Malaya 2: 210, 216. 1973; H. Keng, Concise Fl. Singapore: 49. 1990; M. Turner, Gard. Bull. Singapore 47(1): 263. 1995.
≡ Discostigma rostratum Hassk., Flora 25(2, Beibl.): 33. 1842. Type. lectotype (designated here), Indonesia, Java, s.d., *J. E. Teijsmann 1868* (originally ‘‘Teysmann’’ on the label), P digital image! [P04700642] (Fig. 9A); isolectotypes: K! [K000677710], P digital image! [P04700644].
= Garcinia brevirostris Scheff., Natuurk. Tijdschr. Ned.-Indië 31: 353. 1870 et Flora 53(16): 248. 1870; Pierre, Fl. Forest. Cochinch. 1(5): 5. t. 91C. 1883; Vesque in A. DC. & C. DC., Monogr. Phan. 8: 362. 1893; Merr., Enum. Philipp. Fl. Pl. 3: 83. 1923; Maheshw., Bull. Bot. Surv. India 6: 120. t. 2. fig. 13. 1964; Corner & Watan., Ill. Guide Trop. Pl.: t. 187. 1969; N. P. Singh in B. D. Sharma & Sanjappa, Fl. Ind. 3: 106. 1993; S. Gardner, P. Sidisunthorn & Chayam., Forest Trees S. Thailand 1: 350. fig. 538. 2015; N. Mohanan et al., Rheedea 33(3): 153. 2023. Type. lectotype (designated here), Indonesia, Sumatra, Bangka, Djeboes, fl., s.d., *J. E. Teijsmann s.n.* (originally ‘‘Teysmann’’ on the label), BO digital image! [BO0116415]; isolectotypes: A digital image! [A00067478], K! [K000677711] (Fig. 9B), L digital image! [L0012177, L0012178, U1208258], syn. nov.
= Garcinia eugeniifolia Wall. [Numer. List: 171. Wallich Cat. 4873. 1831, as Garcinia ? ? euginifolia, *nom. nud.*] ex T. Anderson in Hook. f., Fl. Brit. India 1(2): 268. 1874 [as G. eugeniæfolia]; Pierre, Fl. Forest. Cochinch. 1(5): 6. t. 91E, F. 1883 [as G. eugeniæfolia]; King, J. Asiat. Soc. Bengal, Pt. 2, Nat. Hist. 59(2): 150. 1890 [as G. eugeniæfolia]; Engl. in Engl. & Prantl, Die Naturlichen Pflanzenfamilien 3(6): 236. 1893 [as G. eugeniaefolia]; Vesque, Epharmosis 2: 15. t. 99. 1889 et in A. DC. & C. DC., Monogr. Phan. 8: 343. 1893 [as G. eugeniæfolia]; Merr., Philipp. J. Sci. 3: 363. 1908 [as G. eugeniaefolia]; Ridl., Fl. Malay Penins. 1: 169. 1922 [as G. eugeniæfolia]; Maheshw., Bull. Bot. Surv. India 6: 120. 1964; Kochummen & Whitmore, Gard. Bull. Singapore 26(2): 277. 1973 [as G. eugeniaefolia]; Whitmore in Whitmore, Tree Fl. Malaya 2: 210. 1973 [as G. eugeniaefolia]; H. Keng, Concise Fl. Singapore: 48. 1990 [as G. eugeniaefolia]; M. Turner, Gard. Bull. Singapore 47(1): 261. 1995; N. Mohanan et al., Rheedea 33(3): 153. 2023. Type. lectotype [designated by Maheshwari (1964)], Peninsular Malaysia, Penang, fl., 1822, *Wallich Cat. 4873*, CAL digital image! [CAL0000065120]; isolectotypes: G digital image! [G00458408]; K! [K001104113] (Fig. 10A).
= Garcinia calophylla Pierre, Fl. Forest. Cochinch. 1(5): 36. 1883; Vesque in A. DC. & C. DC., Monogr. Phan. 8: 364. 1893; Backer & Bakh. f., Fl. Java (Spermatoph.) 1: 388. 1963. Type. lectotype (designated here), Indonesia, Java, cultivated in Hort. Bot. Bogor., fl. & fr., 1881, *Treub s.n.* (*Herb. L. Pierre 4632*), P digital image! [P04700186] (Fig. 10B); isolectotype: P digital image! [P04700187], syn. nov.
= Garcinia wrayi King, J. Asiat. Soc. Bengal, Pt. 2, Nat. Hist. 59(2): 152. 1890; Vesque in A. DC. & C. DC., Monogr. Phan. 8: 343. 1893; Kochummen & Whitmore, Gard. Bull. Singapore 26(2): 277. 1973. Type. lectotype (designated here), Peninsular Malaysia, Perak, Gunong Batu Pateh, 4,500 feet alt., fl., s.d., *L. Wray 267*, CAL digital image! [CAL0000005862] (Fig. 11A); isolectotypes: CAL digital image! [CAL0000005861], SING! [SING0067963], P digital image! [P04701169]).
= Garcinia gitingensis Elmer, Leafl. Philipp. Bot. 3(55): 1053. 1911. Type. lectotype (designated here), Philippines, Island of Sibuyan, Province of Capiz, Magallanes (Mt. Giting-giting), ♂ fl., May 1910, *A. D. E. Elmer 12526*, A digital image! [A00067528] (Fig. 11B); isolectotypes: E digital image! [E00691513], L digital image! [L2403620, U0111082], MO digital image! [MO934131], NY digital image! [NY71370], US digital image! [US00516746], W digital image! [W1912-0000963]).

##### Description.

***Habit*** trees, 5–20 m tall, 30–100 cm GBH, sometimes buttressed near the base of the stem in large trees; exudate white, turning creamish white, sticky; branchlets green, 4-angular, glabrous. ***Bark*** brown or greyish brown, slightly rough or scaly; inner bark pale brown. ***Leaves*** lanceolate, elliptic or narrowly elliptic, 5.3–11.3 × 2–5.7 cm, apex tapering to a long blunt tip, (0.7–) 1–2 cm long, base cuneate, margin entire or repand, coriaceous, dark green above, paler below, glabrous on both surfaces, midrib slightly raised above, raised below, secondary veins 15–19 on each side, 2.5–5.5 mm apart from each other, departing from the midrib at an angle of 65°–80°, curving towards the margin and connected in distinct loops and united into an intramarginal vein, visible on both surfaces, with intersecondary veins, 1.5–2 mm apart from secondary veins, veinlets reticulate, faint above, visible below, interrupted long wavy lines present, of differing lengths, nearly parallel to the midrib, running across the secondary veins to the apex, visible below; petiole 0.3–1 cm long, 1–1.4 mm diam., grooved above, glabrous, with a small basal appendage clasping the branchlet; fresh leaves tough (not brittle) when crushed; young leaves red or pale greenish red, turning pale green, glossy. ***Inflorescences*** terminal or axillary, in fascicles of 2–10-flowered cymes or solitary (number of flowers per inflorescence of male inflorescences more than female inflorescences). ***Flowers*** unisexual; bracteoles 2, opposite; sepals and petals decussate, concave, glabrous. ***Flower buds*** green, subglobose or globose, 2–3 mm diam. ***Male flowers*** in fascicles of 3–10 flowers, lightly fragrant, 5–6.5 mm diam., sticky; bracteoles triangular, 0.5–1.2 × 0.5–1.1 mm, apex acute; pedicel green, 2–4 mm long, 0.7–1.3 mm diam., glabrous; sepals 4, pale green, the outer pair smaller than the inner pair, the outer pair semi-orbicular, 0.7–1 × 0.6–1.4 mm, apex obtuse, the inner pair broadly ovate or ovate, 1.5–3.5 × 1–3 mm, apex obtuse; petals 4, pale yellow or yellow, broadly elliptic, elliptic or orbicular, 1.5–3.5 × 1–3 mm, subequal, apex obtuse or rounded; stamens numerous, united into 4 bundles, each bundle 1.5–3 × 1–1.7 mm; filaments very short; anthers small; pistillode mushroom-shaped, 2–2.5 mm long; sterile stigma sessile, convex, 1.2–2 mm diam., papillate. ***Female flowers*** in fascicles of 2–3 flowers or solitary, 4.5–7 mm diam.; bracteoles triangular, 0.5–1.2 × 0.5–1 mm, apex acute; pedicel green, 3–6.5 mm long, 1–1.5 mm diam., glabrous; sepals 4, pale green, the outer pair smaller than the inner pair, the outer pair semi-orbicular, 0.7–1.2 × 0.7–1.5 mm, apex obtuse, the inner pair broadly ovate or ovate, 1.7–3.5 × 1–2.5 mm, apex obtuse; petals 4, pale yellow or yellow, broadly elliptic or orbicular, 1.7–4 × 1.8–3.5 mm, subequal, apex rounded or obtuse; staminodes absent; pistil mushroom-shaped; ovary pale green, subglobose or globose, 1–2 × 1.3–2.3 mm; stigma pale yellow, sessile, convex, 2.5–3 mm diam., unlobed, papillate. ***Fruits*** berries, subglobose or globose, 1–1.2 × 0.7–1.2 cm, green (colour of ripe fruits not recorded), smooth and glabrous, pericarp coriaceous, c. 6–6.5 mm thick, cut fruits with a sticky white exudate, with persistent sepals; persistent stigma blackish brown or dark brown, discoid and shallowly concave, 7–9 mm diam., unlobed, papillate; fruiting stalk 3–6.5 mm long, 1.2–2 mm diam. ***Seeds*** 1–2, reddish brown or dark brown, depressed subglobose, 0.9–1.2 × 0.7–0.9 cm, with a thin fleshy pulp.

##### Distribution.

**Figure 12.**
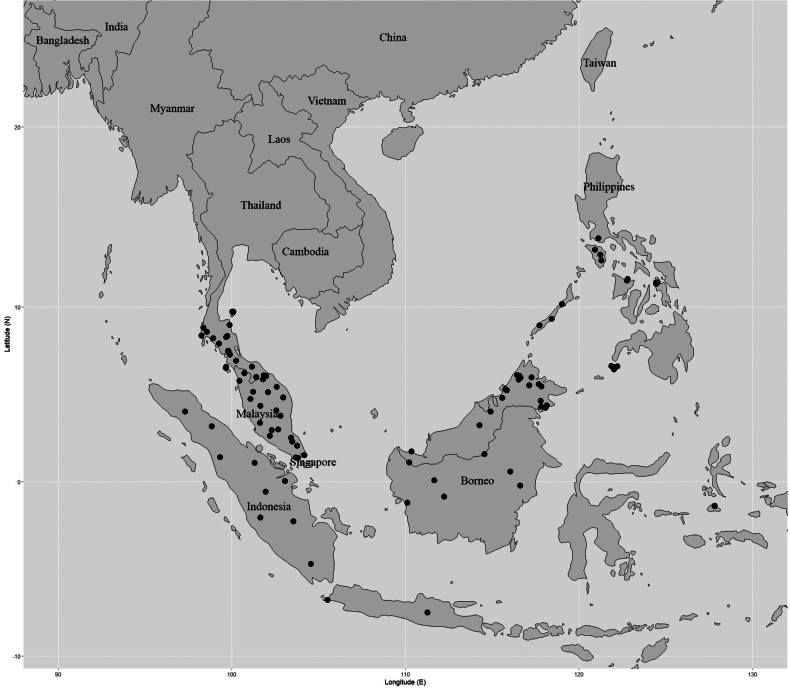
Distribution of *Garcinia rostrata*. It is known from Peninsular Thailand and Peninsular Malaysia to Borneo and the Philippines. In Thailand, this species is found only in the peninsular region. Map: Pichet Chonton and Chatchai Ngernsaengsaruay.

Peninsular Thailand, Peninsular Malaysia [Kedah (including Langkawi Island), Penang, Perak, Kelantan, Terengganu, Pahang, Selangor, Negeri Sembilan, Malacca, Johor], Singapore, Indonesia (Sumatra, Java, Maluku Islands), Borneo [Malaysia (Sarawak, Sabah), Indonesia (Kalimantan)], Philippines (Mindoro, Capiz, Leyte, Palawan, Basilan) (Fig. 12).

##### Distribution in Thailand.

**Peninsular**: Surat Thani, Phangnga, Nakhon Si Thammarat, Trang, Satun, Songkhla, Pattani, Yala, Narathiwat (Fig. 12).

##### Habitat and ecology.

This species is found in tropical evergreen rain forests, lower montane rain forests, and secondary forests, sometimes along streams, at elevations of 0–1,500 m a.m.s.l.

##### Phenology.

Flowering and fruiting more than once, nearly throughout the year, with a peak from February to May.

##### Conservation status.

*Garcinia rostrata* is widely distributed from Peninsular Thailand to Borneo and the Philippines. It is known from many localities and has a large EOO of 3,582,193.06 km^2^ and an AOO of 180 km^2^. In Thailand, this species is known to be naturally distributed only in the peninsular region and has an EOO of 88,529.82 km^2^ and an AOO of 76 km^2^. Because of its wide distribution and the number of localities and because there does not appear to be an imminent threat to the plants or their habitats. Therefore, we consider the conservation assessment here as LC in agreement with de Kok (2024b).

##### Etymology.

The specific epithet of *Garcinia rostrata* is a Latin word meaning beaked, with a long beak, and refers to the fact that the leaf apex has a long beak.

##### Vernacular names.

Dan mi (ดันหมี) (Narathiwat, from the specimen *C. Phengklai & C. Niyomdham 8001*); Nuan (นวล) (Nakhon Si Thammarat, from the specimen *A. F. G. Kerr 15683*); **Nuan khao** (**นวลขาว**) (Surat Thani, from the specimen *Put 770*); Nuan dong (นวลดง) (Surat Thani, from the specimen *Put 788*); Nuan daeng (นวลแดง) (Surat Thani, from the specimen *Put 1163*); Muang lai (มวงลาย) (Surat Thani, from the specimen *P. Suvarnakoses 837*).

##### Uses.

The fruits of *Garcinia rostrata* serve as a food source for wildlife.

##### Lectotypifications.

*Discostigma rostratum* was named by Hasskarl (1842: 33) and was transferred to the genus *Garcinia* by Miquel (1864: 209), who cited the type locality and the collector’s name “Bali (*Teysmann*)”. He did not select a holotype, nor did he mention the collector number and the name of the herbarium where the material was kept. However, we located only the *Teysmann 1868* material collected from Java (originally “E Java” on the label) at K [K000677710] and at P [P04700642, P04700644], and following Art. 9.6 of the ICN (Turland et al. 2018), they constitute syntypes. Bali is a province of Indonesia and located east of Java, but the type locality appears to be East Java, so perhaps there has been a transcription error in the original publication. Therefore, the P [P04700642] specimen is considered here as the lectotype, following Arts. 9.3 and 9.12 of the ICN (Turland et al. 2018).

*Garcinia wrayi* was named by King (1890: 152–153), who cited four gatherings, *Wray 267, 362, 1527*, and *Scortechini 3235*, collected from Perak, “Gunong Batu Pateh and Ulu Batang Padang, at elevations of 4,500 feet and upwards”. He did not mention the name of the herbaria where the materials were housed, and following Art. 9.6 of the ICN (Turland et al. 2018), these are syntypes. We located the materials *L. Wray 267* (Gunong Batu Pateh, 4500 feet alt.) at CAL [CAL0000005861, CAL0000005862], SING [SING0067963], and P [P04701169]; *L. Wray 362* (summit of Gunong Batu Pateh, 6700 feet alt.) at CAL [CAL0000005859], SING [SING 0067962], and P [P04701168]; *L. Wray 1527* (Ulu Batang Padang, 4900 feet alt.) at CAL [CAL0000005863]; and *R. F. Scortechini 3236* (locality unspecified) at CAL [CAL0000005860]. The specimen number appears to be *R. F. Scortechini 3236*, so perhaps there has been a transcription error in the original protologue. George King was a superintendent of Calcutta Botanic Gardens (1871–1898) and a director of Botanical Survey of India (1891–1898) (Stafleu and Cowan 1979). Therefore, the material *L. Wray 267* at CAL [CAL0000005862] is better preserved and more complete than the others and is designated here as the lectotype, following Arts. 9.3 and 9.12 of the ICN (Turland et al. 2018).

*Garcinia brevirostris* was named by Scheffer (1870a: 353; 1870b: 248), who cited the type locality and the collector’s name “Bangka, prope Müntok et Djeboes (*Teysmann*)”. He did not select a holotype, nor did he mention the collector number and the name of the herbaria where the material was deposited. However, we found two sets of material: (1) *Teysmann s.n.* collected from Bangka, Djeboes at A [A00067478], BO [BO0116415], K [K000677711], and L [L0012177, L0012178, U1208258] and (2) *Teysmann s.n.* collected from Bangka, prope Müntok at L [L2408901, U1208257], and following Art. 9.6 of the ICN (Turland et al. 2018), they constitute syntypes. Rudolph Herman Christiaan Carel Scheffer (1844–1880) was a Dutch botanist and a director of the Botanic Gardens Buitenzorg (now known as Bogor) (1868–1880) (Stafleu and Cowan 1985). Therefore, the BO [BO0116415] specimen is in the best condition and is selected here as the lectotype, following Arts. 9.3 and 9.12 of the ICN (Turland et al. 2018).

*Garcinia gitingensis* was named by Elmer (1911: 1053), who cited three gatherings: *A. D. E. Elmer 12213, 12482*, and *12526* collected from Magallanes (Mt. Giting-giting), Province of Capiz, Island of Sibuyan. He did not mention the name of the herbaria where the specimens were kept, and following Art. 9.6 of the ICN (Turland et al. 2018), these are syntypes. We traced the specimens *A. D. E. Elmer 12213* at E [E00691511], L [L2403621, U1208223], and W [W1912-0000852]; *A. D. E. Elmer 12482* at E [E00691512], GH [GH00067529], MO [MO716956], NY [NY71369], US [US00114336], and W [W1912-0000851]; and *A. D. E. Elmer 12526* at A [A00067528], E [E00691513], L [L2403620, U0111082], MO [MO934131], NY [NY71370], US [US00516746], and W [W1912-0000963]. Adolph Daniel Edward Elmer (1870–1942) was an American botanist who collected exclusively in Washington, California, Borneo, and the Philippines. His type specimens were housed at A and PNH (Stafleu and Cowan 1976). Hence, the specimen *A. D. E. Elmer 12526* at A [A00067528] is in the best condition and clearly shows the diagnostic characters for the species and is selected here as the lectotype, following Arts. 9.3 and 9.12 of the ICN (Turland et al. 2018).

Pierre (1883: 37) established *Garcinia calophylla* based on the material collected from Java, and it was cultivated in le jardin botanique de Bogor (originally “Plante cultivée dans le jardin bot. de Buitenzorg à Java” in the first publication). He did not mention the collector number and the name of the herbarium where the material was kept. However, we located the material *Treub s.n.* (*Herb. L. Pierre 4632*) cultivated in Hort. Bot. Bogor. (Horto Botanico Bogoriensi) at P [P04700186, P04700187], and following Art. 9.6 of the ICN (Turland et al. 2018), it constitutes a syntype. Therefore, the P [P04700186] specimen is in the best condition and is chosen here as the lectotype, following Arts. 9.3 and 9.12 of the ICN (Turland et al. 2018).

##### Note.

Maheshwari (1964) considered the specimen *Wallich Cat. 4873* at CAL [without barcode] as the lectotype of *Garcinia eugeniifolia*, without isolectotypes. However, we located the lectotype at CAL with barcode [CAL0000065120] and isolectotypes at G [G00458408] and K [K001104113].

##### Additional specimens examined.

**Thailand. Peninsular**: • Surat Thani [Ko Phangan, ♂ fl., 3 Jun 1927, *Put 770* [BK (P. F. Stevens det. as G. cf. merguensis and *G. brevirostris*), BM (as *Garcinia* sp.), C, K, L [L2416507], and P [P05061541] (as *G. merguensis*)]; • ibid., fr., 5 Jun 1927, *Put 788* [BM (as *Garcinia* sp.), K (as *G. merguensis*)]; • ibid., fr., 4 Nov 1927, *Put 1159* [BK (P. F. Stevens det. as G. cf. merguensis and *G. brevirostris*), K (as *G. merguensis*)]; • ibid., fr., 4 Nov 1927, *Put 1163* [BK (P. F. Stevens det. as G. cf. merguensis and *G. brevirostris*), BM (as *Garcinia* sp.), K (as *G. merguensis*)]; • ibid., fr., 4 Dec 1974, *R. Geesink et al. 7751* [AAU (C. Ngernsaengsaruay det. as *G. rostrata*), BKF, C, and P [P05061701] (as *Garcinia* sp.)]; • Locality unspecified, young fr., 6 Aug 1955, *P. Suvarnakoses 837* (BKF) (as *G. rostrata*); • Locality unspecified, ♂ fl., 25 Jun 1975, C. *Promdej et al 226* [AAU (C. Ngernsaengsaruay det. as *G. rostrata*), BKF (as *G. merguensis*), C (as *Garcinia* sp.), K (H. Toyama det. as *G. merguensis*), P [P05062021] (as *Garcinia* sp.); • Ko Phangan, ♂ fl. (spirit collection), 25 Jun 2024, *C. Ngernsaengsaruay & P. Chanton G71-25062024* (BKF); • ibid., fr. (spirit collection), 3 Feb 2025, *C. Ngernsaengsaruay & P. Chanton G72-03022025* (BKF); • Phaeng Noi Waterfall nature trail, Than Sadet-Ko Phangan National Park, sterile, 30 Mar 2025, *C. Ngernsaengsaruay* personal observation with photos]; • Phangnga [Takua Pa District, fr., 12 May 1968, *C. F. van Beusekom & C. Phengkhlai 727* [AAU (C. Ngernsaengsaruay det. as *G. rostrata*), BKF (as *G. merguensis*), C, K, and P [P05062007] (*Garcinia* sp.); • Ton Pariwat Wildlife Sanctuary, fl., 24 Apr 2006, *S. Gardner & P. Sidisunthorn ST2614* [BKF and K (as *Garcinia* sp.)]; • Thai Mueang Beach, fl., 3 Apr 2008, *C. Maknoi 2423* (QBG) (as *Garcinia* sp.); • Krabi [Khao Pra Bang Khram Wildlife Sanctuary, Khlong Thom Nuea Subdistrict, Khlong Thom District, 18 Mar 2018, *C. Ngernsaengsaruay* personal observation with photos; • Khao Phanom Bencha National Park, Thap Prik Subdistrict, Mueang District, 14 Feb 2022, *C. Ngernsaengsaruay & W. Boonthasak G68-14022022* (BKF)]; • Nakhon Si Thammarat [Sichon District, fr., 12 May 1928, *A. F. G. Kerr 15683* [BK (P. F. Stevens det. as G. cf. merguensis and *G. brevirostris*), BM (as *Garcinia* sp.), C, K, L [L2416493], and P [P05061542] (as *G. merguensis*)]; • Karom Waterdall, Khao Luang National Park, Lan Saka District, young fr., 24 Nov 1984, *J. F. Maxwell 84-487* [BKF, P [P04701380], and PSU (as *G. eugeniaefolia*)]; • Khao Men, young fr., 10 Mar 1957, *Snan 996* (BKF) (as *G. rostrata*)]; • Trang [Khao Chong, fl., 25 Jan 1958, *T. Smitinand 4099* (BKF) (as *G. rostrata*); • Khao Banthat, Yan Ta Khao District, ♂ fl., 5 Apr 2003, *D. J. Middleton et al. 1892* (A [00466329] and BKF) (as *Garcinia* sp.)]; • Satun [Tarutao National Park, fl., 27 Feb 1966, *B. Hansen & T. Smitinand 12468* [BKF (as *G. eugeniaefolia*), C and K (as *Garcinia* sp.)]; • ibid., fl., 16 Feb 2005, *S. Gardner et al. ST1544* [BKF and K (as *Garcinia* sp.); • Ton Te Waterfall nature trail, Khao Banthat Wildlife Sanctuary, Palian District, sterile, 10 Jul 2016, *C. Ngernsaengsaruay G67-10072016* (BKF); • Khao Chong, Chong Subdistrict, Na Yong District, 16 Feb 2022, *C. Ngernsaengsaruay* personal observation with photos; • Sai Rung Waterfall nature trail, Khao Banthat Wildlife Sanctuary, Yan Ta Khao District, 16 Feb 2022, *C. Ngernsaengsaruay & W. Boonthasak G69-16022022* (BKF)]; • Songkhla [Ton Nga Chang Wildlife Sanctuary, fr., 27 Jan 1992, *P. Puudjaa 58* (BKF) (as *G. rostrata*); • Ton Nga Chang Waterfall, fl., 14 May 2004, *S. Gardner & P. Sidisunthorn ST0516* [BKF and K (as *Garcinia* sp.)]; • Pattani [Khao Kala Khiri, fr., 2 Apr 1928, *A. F. G. Kerr 14978* [BK (P. F. Stevens identified as G. cf. merguensis and *G. brevirostris*), C (C. Ngernsaengsaruay det. as *G. merguensis*, 31 Jul 2018), K, L [L2416492], and P [P05061543] (as *G. merguensis*)]; • Yala [Bang Lang National Park, Aiyoeweng Subdistrict, Betong District, ♂ fl., 23 May 2022, *C. Ngernsaengsaruay G70-23052022* (BKF)]; • Narathiwat [Chat Warin Waterfall, fl., 15 Jun 1970, *T. Smitinand 10975* [BKF, C, K, and P [P05062037] (as *Garcinia* sp.)]; • To Daeng Peat Swamp Forest, Su-Ngai Kolok District, fr., 25 Sep 1992, *C. Phengklai & C. Niyomdham 8001* [BKF, K, and P [P 06899611, P06899612] (as *Garcinia* sp.)]; • ibid., fr., Aug 1993, *C. Niyomdham s.n.* (BKF) (as *Garcinia* sp.); • ibid., fr., 10 Apr 1997, *C. Niyomdham 4983* [AAU (C. Ngernsaengsaruay det. as *G. rostrata*), BKF (as *Garcinia* sp.)]; • Sukhirin District, ♀ fl., 22 Jul 2003, *P. Puudjaa 1180* (BKF) (as *Garcinia* sp.); • Chat Warin Waterfall, Budo-Su-ngai Padi National Park, 8 Feb 2025, C. Ngernsaengsaruay & P. Chanton personal observation with photos].

#### ﻿4. Garcinia santisukiana

Taxon classificationPlantaeMalpighialesClusiaceae

Ngerns. & Suddee, Kew Bull. 77: 121. figs 1, 2. 2022.

8CE04944-33BB-5A72-85A5-0AD2500D27FB

##### Type.

***Holotype***, • Thailand, Ubon Ratchathani Province, Khong Chiam District, Na Pho Klang Subdistrict, Pha Taem National Park, Dong Na Tham Forest, in dry evergreen forest, 420 m alt., ♀ fl. & fr., 23 Sep 2020, *C. Ngernsaengsaruay & W. Surawoot G02-23092020*, BKF!; ***isotypes***: A!, K!.

##### Description.

**Figure 13.**
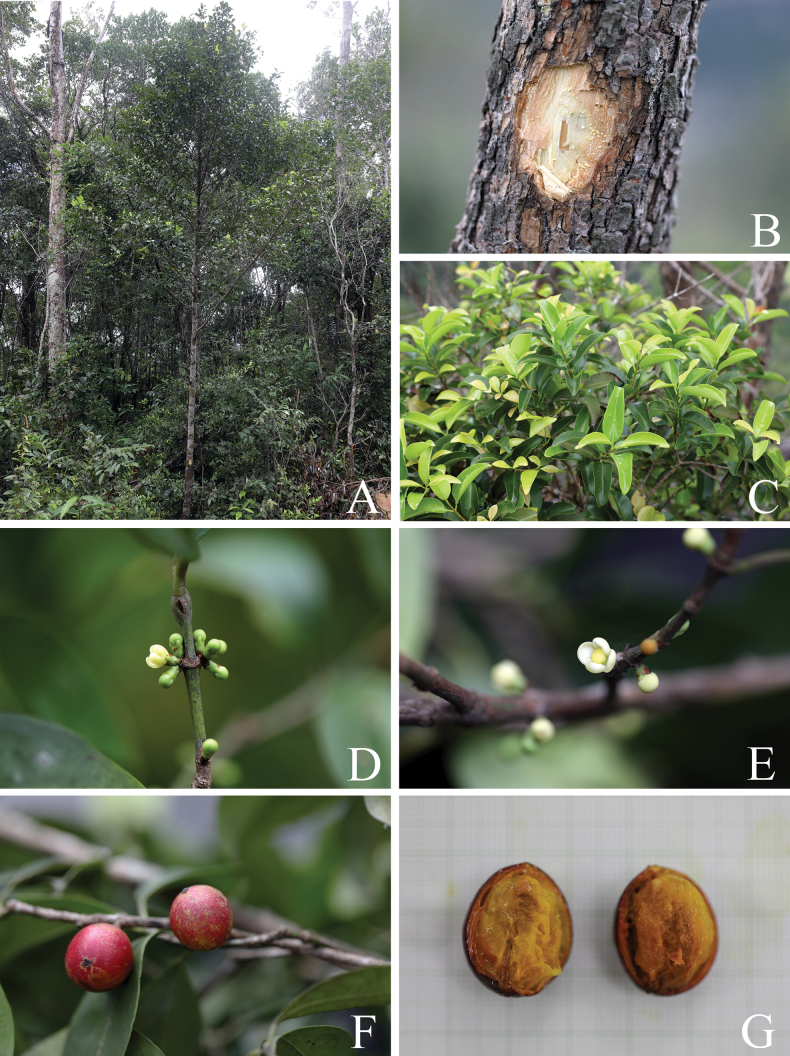
*
Garcinia santisukiana*. A. Habit and habitat; B. Slashed bark with pale yellow exudate; C. Branchlets, mature and young leaves; D, E. Branchlets and inflorescences with female flowers; F. Branchlets and ripe fruits; G. Fruits and seeds with fleshy pulp. Photos: Chatchai Ngernsaengsaruay.

***Habit*** trees, 5–18 m tall, 20–85 cm GBH; exudate pale yellow, sticky; branchlets 4-angular, glabrous. ***Bark*** greyish-brown to dark brown, scaly; inner bark pale yellow. ***Leaves*** elliptic or obovate, 2.7–9.7 × 1.5–4.3 cm, apex acute, sometimes retuse, base cuneate, margin entire, subcoriaceous or coriaceous, dark green above, paler below, glabrous on both surfaces, midrib flattened above, raised below, secondary veins 9–14 on each side, curving towards the margin and connected in distinct loops and united into an intramarginal vein, visible on both surfaces, with intersecondary veins, veinlets reticulate, faint on both surfaces, with scattered black gland dots on both surfaces, interrupted long wavy lines present, of differing lengths, nearly parallel to the midrib, running across the secondary veins to the margin, visible below; petiole 0.5–1.3 cm long, 1–1.7 mm diam., shallowly grooved above, glabrous, with a small basal appendage clasping the branchlet; fresh leaves brittle when crushed; young leaves red or reddish brown, turning pale green, glossy. ***Inflorescences*** on branchlets at leafless nodes, in fascicles of 3–5 flowered cymes or solitary. ***Flowers*** unisexual, lightly fragrant, 4–7 mm diam.; bracteoles 4, decussate, green; sepals and petals decussate, sepals green, glabrous, petals creamish white or pale yellow. ***Flower buds*** pale green, subglobose or globose, 2.5–3.5 mm diam. ***Male flowers*** mostly in fascicles of 3–5 flowers; bracteoles triangular, 0.3–0.7 × 0.6–1 mm, apex acute; pedicel green, 1–2 mm long, 1–1.7 mm diam., glabrous; sepals 4, semi-orbicular, c. 1 × 1–1.5 mm, apex rounded; petals 4, obovate, 3–5 × 2.2–3.2 mm, concave, apex rounded; stamens numerous, united into 4 bundles, each bundle 2–3 × 1.2–1.5 mm, creamish white; filaments very short; anthers small; pistillode mushroom-shaped, 3–3.6 mm long; sterile stigma pale yellow, sessile, convex, 1–1.5 mm diam., papillate. ***Female flowers*** solitary or in fascicles of 3–5 flowers; bracteoles semi-orbicular, 0.8–1 × 1–1.5 mm, apex rounded; pedicel green, 1.5–2.5 mm long, 1.5–1.8 mm diam., glabrous; sepals 4, equal, semi-orbicular, 1–1.5 × 1–2 mm, apex rounded; petals 4, suborbicular or obovate, 3–4 × 2.5–3.5 mm, concave, apex rounded; staminodes united into 4 bundles at the base of ovary, opposite petals; pistil mushroom-shaped; ovary green, subglobose, 1–2 × 1.8–2.2 mm; stigma pale yellow, sessile, convex, 2–2.2 mm diam., weakly or shallowly 4-lobed, papillate. ***Fruits*** berries, subglobose or ovoid, 1.5–2.7 × 1–2.5 cm, green, turning red when ripe, smooth and glabrous, pericarp coriaceous, c. 0.8 mm thick, cut fruits with a sticky yellow exudate, with small persistent sepals; persistent stigma blackish brown, flattened, 2–2.2 mm diam., weakly or shallowly 4-lobed, papillate; fruiting stalk 1.5–2.5 mm long. ***Seeds*** 1–2, brown mottled with pale brown, compressed, one side flat with conspicuous hilum, another side slightly convex, elliptic or oblong in outline, 1.5– 2 × 1–1.5 cm, rounded at both ends, with a yellow fleshy pulp (Fig. 13).

##### Distribution.

Known only from north-eastern and eastern regions of Thailand, but to be expected in Laos and Cambodia.

##### Distribution in Thailand.

**North-Eastern**: Buengkan, Nakhon Phanom; **Eastern**: Si Sa Ket, Ubon Ratchathani.

##### Habitat and ecology.

It is found in dry evergreen forests and deciduous dipterocarp forests on sandstone crevices, at elevations of 150–600 m a.m.s.l.

##### Phenology.

Flowering and fruiting more than once, flowering August to December, fruiting September to February and June.

##### Conservation status.

*Garcinia santisukiana* is known only from four provinces of Thailand, but to be expected in Laos and Cambodia. The species is known from small populations in the type and non-type localities, which lie within protected areas. It has an EOO of 29,848.67 km^2^ and a small AOO of 20 km^2^. Because of this species with narrow geographical range and the number of locations, we consider the conservation assessment here as VU [B2a,b(iii)].

##### Etymology.

The specific epithet of *Garcinia santisukiana* was in honour of the late Prof. Dr Thawatchai Santisuk (1944–2020), one of Thailand’s most widely respected plant taxonomists (Ngernsaengsaruay and Suddee 2022).

##### Vernacular names.

**Nuan santisuk** (**นวลสันติสุข**); Yang ueng (ยางอึ่ง) (Ubon Ratchathani, local people around Dong Na Tham Forest) (Ngernsaengsaruay and Suddee 2022).

##### Uses.

The fleshy pulp surrounding the seeds is edible and has a sweet and sour taste.

##### Notes.

The morphological characters and data reported here for this species were mostly taken from Ngernsaengsaruay and Suddee (2022).

In addition to Ngernsaengsaruay and Suddee (2022), the natural distribution in Buengkan, Nakhon Phanom, and Si Sa Ket Provinces is newly recorded here.

According to Ngernsaengsaruay and Suddee (2022), the conservation status of *Garcinia santisukiana* was proposed as LC. In this study, because of this species with a narrow geographical range and a small number of locations, we therefore suggest the conservation status VU [B2a,b(iii)].

##### Additional specimens examined.

**Thailand. North-Eastern**: • Buengkan [Chet Si Waterfall, Phuwua Wildlife Sanctuary, Ban Tong Subsistrict, Seka District, fr., 7 Jun 2008 (as *Garcinia* sp.), *T. Wongprasert 086-19* (BKF)]; • Nakhon Phanom [Tat Kham Waterfall, Phu Langka National Park, Ban Phaeng District, fl., 25 Aug 2001 (as *Garcinia* sp.), *R. Pooma et al. 2646* (BKF)]; **Eastern**: • Si Sa Ket [Khao Phanom Dong Rak (originally “Dongrak Range at Chong Bat Lak” on the label), Kantharalak District, ♂ fl., 16 Aug 1976 (as G. cf. merguensis and *G. brevirostris*, det. P. F. Stevens, Sep 1997), *J. F. Maxwell 76-515* (AAU, BKF); • Ubon Ratchathani [Pha Chana Dai, Pha Taem National Park, fr., 28 Feb 2007 (as *Garcinia* sp.), *S. Suddee et al. 3075* (paratype BKF); • ibid., fl., 7 Nov 2018, *S. Suddee et al. 5393* (paratype BKF); • ibid., ♂ fl., 10 Dec 2020, *C. Ngernsaengsaruay & W. Surawoot G05-10122020* (paratypes BKF, K); • Dong Na Tham Forest, Pha Taem National Park, Na Pho Klang Subdistrict, Khong Chiam District, ♀ fl. & fr., 23 Sep 2020, *C. Ngernsaengsaruay & W. Surawoot G03-23092020* (paratypes A, BKF, K); • ibid., fr., 23 Sep 2020, *C. Ngernsaengsaruay & W. Surawoot G04-23092020* (paratypes BKF)].

## Supplementary Material

XML Treatment for Garcinia sect. Discostigma

XML Treatment for Garcinia merguensis

XML Treatment for Garcinia minutiflora

XML Treatment for Garcinia rostrata

XML Treatment for Garcinia santisukiana

## ﻿Additional information

### Use of AI

No use of AI was reported.

### Author ORCIDs

Chatchai Ngernsaengsaruay https://orcid.org/0000-0002-7131-976X

Pichet Chanton https://orcid.org/0009-0001-7325-6109

### Data availability

All of the data that support the findings of this study are available in the main text.
